# Test-Retest Variability and Discriminatory Power of Measurements From Microperimetry and Dark Adaptation Assessment in People With Intermediate Age-Related Macular Degeneration – A MACUSTAR Study Report

**DOI:** 10.1167/tvst.12.7.19

**Published:** 2023-07-21

**Authors:** Bethany E. Higgins, Giovanni Montesano, Hannah M. P. Dunbar, Alison M. Binns, Deanna J. Taylor, Charlotte Behning, Amina Abdirahman, Matthias C. Schmid, Jan H. Terheyden, Nadia Zakaria, Stephen Poor, Robert P. Finger, Sergio Leal, Frank G. Holz, Gary S. Rubin, Ulrich F. O. Luhmann, David P. Crabb, on behalf of the MACUSTAR Consortium

**Affiliations:** 1 City, University of London, London, UK; 2 Moorfields Eye Hospital NHS Foundation Trust, London, UK; 3 UCL Institute of Ophthalmology, London, UK; 4 Institute of Medical Biometry, Informatics and Epidemiology, Medical Faculty, University of Bonn, Bonn, Germany; 5 Department of Ophthalmology, University Hospital Bonn, Bonn, Germany; 6 Department of Translational Medicine, Novartis Institute for Biomedical Research, Cambridge, MA, USA; 7 Department of Ophthalmology Research, Novartis Institute for Biomedical Research, Cambridge, MA, USA; 8 Bayer AG, Berlin, Germany; 9 Roche Pharmaceutical Research and Early Development, Translational Medicine Ophthalmology, Roche Innovation Center Basel, Basel, Switzerland

**Keywords:** test-retest, repeatability, macular integrity assessment (S-MAIA), AdaptDx, age-related macular degeneration (AMD)

## Abstract

**Purpose:**

The purpose of this study was to assess test-retest variability and discriminatory power of measures from macular integrity assessment (S-MAIA) and AdaptDx.

**Methods:**

This is a cross-sectional study of 167 people with intermediate age-related macular degeneration (iAMD), no AMD (controls; *n* = 54), early AMD (*n* = 28), and late AMD (*n* = 41), recruited across 18 European ophthalmology centers. Repeat measures of mesopic and scotopic S-MAIA average (mean) threshold (MMAT decibels [dB] and SMAT [dB]) and rod intercept time (RIT [mins]) at 2 visits 14 (±7) days apart were recorded. Repeat measures were assessed by Bland-Altman analysis, intra-class correlation coefficients (ICCs) and variability ratios. Secondary analysis assessed the area under the receiver operating characteristic curves (AUC) to determine the ability to distinguish people as having no AMD, early AMD, or iAMD.

**Results:**

Data were available for 128, 131, and 103 iAMD participants for the mesopic and scotopic S-MAIA and AdaptDx, respectively. MMAT and SMAT demonstrate similar test-retest variability in iAMD (95% confidence interval [CI] ICC of 0.79–0.89 and 0.78–0.89, respectively). ICCs were worse in RIT (95% CI ICC = 0.55–0.77). All tests had equivalent AUCs (approximately 70%) distinguishing between subjects with iAMD and controls, whereas early AMD was indistinguishable from iAMD on all measures (AUC = <55%). A learning effect was not seen in these assessments under the operating procedures used.

**Conclusions:**

MMAT, SMAT, and RIT have adequate test-retest variability and are all moderately good at separating people with iAMD from controls.

**Translational Relevance:**

Expected levels of test-retest variability and discriminatory power of the AdaptDx and MAIA devices in a clinical study setting must be considered when designing future trials for people with AMD.

## Introduction

MACUSTAR is a prospective multicenter clinical study aiming to develop end points for clinical trials in people with age-related macular degeneration (AMD)^1^; it has cross-sectional and longitudinal components with the former designed primarily to assess measurement properties of structural and functional candidate end points. The longitudinal component will evaluate how different measures might track progression in people with intermediate AMD (iAMD). Testing of visual function may elicit additional patient-relevant information, compared to grading scales based on structural appearance alone, and inform structure-functional relationships.^2^ Therefore, one aim of MACUSTAR is to assess a set of functional vision tests that might characterize changes in iAMD before late-stage AMD.^1^

Aside from interest in visual function measures for trials, there is growing investment by clinics in instruments for measuring visual function beyond those using conventional charts. Examples include microperimetry (fundus controlled perimetry) and dark adaptation technology. In the literature, there is evidence to support the role of both mesopic and scotopic microperimetry^3^^,^^4^ in assessing people with AMD especially in research and as study end points.^5^ Moreover, measures of rod-mediated dark adaptation (slower return to retinal sensitivity following a bright light flash stimulus) may provide a sensitive measure of AMD progression.^6^^–^^8^ In this context, a specific aim of MACUSTAR is to evaluate measurements from mesopic and scotopic microperimetry macular integrity assessment (S-MAIA; CenterVue, Padova, Italy) and dark adaptation (AdaptDx; MacuLogix, Middletown, PA, USA) as potential functional biomarkers for iAMD. These instruments are the subject of this study, with the MACUSTAR assessment of other chart-based methods of assessing visual function described in a previous report.^9^

Repeated measurements on the same subject vary around a true value because of measurement error. Confusingly, different terms are used to describe measurement error, including, for example, precision, repeatability, inter-session variability, inter-test variability, and test-retest variability. For simplicity, we will adopt the last. An understanding of the test-retest variability of a measurement, estimated by the difference in two repeated measures recorded over a short period of time, is critical for the clinical use of the measurement or adoption in trials. This must be linked to the minimal clinically significant difference insofar as a “real” change can only be registered if it exceeds the test-retest variability. Assessment of test-retest variability for a device in small numbers of visually healthy people is inadequate. For instance, in a recent systematic review of the measurement of dark adaptation, with a focus on the AdaptDx device,^10^ we found only one study to have adequately attempted to assess test-retest variability and this did not specify the disease status of the cohort recruited.^11^ Better reports on the topic for measurements from S-MAIA exist.^5^^,^^12^^,^^13^ MACUSTAR offers a unique opportunity to estimate test-retest variability of these visual function measures in a large number of people with iAMD; this is the main focus of our study.

We primarily aim to estimate test-retest variability for measurements from mesopic and scotopic assessments using MAIA microperimetry and AdaptDx dark adaptation in eyes with iAMD from the MACUSTAR cross-sectional study. We conduct secondary analyses, including an assessment of how well summary measurements from the devices distinguish people with iAMD from people with “early AMD” and visually healthy controls. We also estimate other measurement properties of the devices, including reliability, participant compliance to complete the examinations, and practice/learning effects. Such data will be particularly useful for implementing these measures of visual function in future iAMD clinical trials.

## Methods

The design of the MACUSTAR study (Registration NCT03349801; www.clinicaltrials.gov) has been described previously^1^^,^^14^ with participants recruited from 20 clinical sites from 7 European countries of which 18 took part in the cross-sectional study part. For the present study, we only extracted data collected from participants in the cross-sectional component of MACUSTAR; this comprised a baseline and a short-term follow-up visit (14 ± 7 days) with at least 150 people with iAMD planned to be recruited. In addition, smaller numbers of people with early AMD, late-stage AMD, and normal ocular aging changes only (controls) were also recruited. The sample size rationale has been described previously.^1^^,^^9^ To deduce the applicability of longitudinal data to different populations and given the strong genetic background of AMD, ethnicity data were collected by self-report. Strengthening the Reporting of Observational Studies in Epidemiology (STROBE) guidelines were adhered to. The Beckman scale^15^ was used to determine AMD status, assessed by a central reading center on the basis of multimodal imaging (color fundus photography, confocal infrared photography, fundus autofluorescence, and spectral-domain optical coherence tomography images) from a dedicated screening visit. Images were graded by a junior reader followed by a senior reader,^16^ which included biomarkers suggestive of disease progression; the detail of this and a full description of inclusion and exclusion criteria are given elsewhere.^9^^,^^17^ No incentives for participation were offered, but travel expenses were reimbursed. All participants gave written informed consent and the study conformed to the Declaration of Helsinki.

At both cross-sectional study visits participants performed tests of visual function, as well as imaging and completed questionnaires. A study eye for each participant was defined as one with the better best corrected visual acuity (BCVA) determined at the screening visit using the Early Treatment of Diabetic Retinopathy Study (ETDRS) chart.

Our focus is solely on the device-based tests of visual function, namely mesopic and scotopic microperimetry (S-MAIA) and dark adaptation (AdaptDx). These device-based tests were performed on the same day after completion of conventional chart-based tests. We assessed data from these devices in participants who successfully had BCVA recorded at two visits. This inclusion criterion makes our results representative of a study population that can adequately perform chart-based visual function assessment.

The device-based visual function testing (S-MAIA followed by AdaptDx) was carried out by certified technicians in accordance with a standard operating procedure.

S-MAIA is a modified version of the macular integrity assessment microperimeter assessing both mesopic testing with achromatic stimuli and dark-adapted two-color scotopic testing with cyan (505 nm) and red (627 nm) stimuli.^18^ Scotopic testing is thought to be more relevant when probing visual dysfunction function in AMD as rods are primarily affected in AMD,^19^ but it is more inconvenient because of the need for dark adaptation.^18^^,^^20^ The study eye was dilated (1% tropicamide) and the participant was dark adapted for 5 minutes prior to beginning the mesopic microperimetry. The participant was positioned on the chin rest (non-study eye occluded) and then instructed to respond (by pressing a button) to stimuli while fixating on a red fixation circle. The technician used the device to determine the optic disc center and the participant's preferred retinal locus was estimated automatically by the S-MAIA in order to correctly center the grid. This study used a customized stimulus grid of 33 points located at 0 degrees, 1 degrees, 3 degrees, 5 degrees, and 7 degrees from fixation.^12^ First, mesopic microperimetry was performed using achromatic stimuli (Goldmann III) presented for 200 ms using a 4-2 staircase strategy with a background luminance of 1.27 cd/m^2^ and an initial target luminance of 2.6 ± 0.5 asb. Next, after a further 30 minutes of dark adaptation, scotopic microperimetry was performed using a red (627 nm) stimulus (Goldmann III) presented for 200 m/s using a 4-2 staircase strategy with no background illumination and an initial target luminance of 0.01 asb. A red filter was in place on the S-MAIA screen during scotopic testing to ensure the participant remained dark adapted throughout testing. The tests were expected to take approximately 5 minutes each.^12^ Both tests used the 33-point test pattern. In addition, the standard operating procedure instructed technicians to use the follow-up mode for both tests for the follow-up visit; ensuring the same retinal locations are examined on retesting, which makes sense in end point-exploring studies. (The follow-up mode on the S-MAIA also shortens test times.) The standard operating procedure also instructed technicians to note if tests failed either of two reliability criteria (fixation losses ≥ 30% or if the 95% bivariate contour ellipse area [BCEA] > 50 degrees^2^). It is important to note that at the baseline visit participants first performed a microperimetry practice session, based on a 9-point grid, done with the aim of mitigating any practice/learning effects.

Dark adaptation assessment, using the AdaptDX, was conducted after the scotopic microperimetry and the participant remained dark adapted. The participant was positioned on the adjustable chin rest (non-study eye occluded) and asked to focus on the red fixation light with the technician aligning the eye to the eye tracker. The participant was advised there would be a bleaching flash followed by a blue-green spot and they were then instructed to press the button when this was seen. Pupil size was automatically assessed by the device to standardize retinal illumination during the testing procedure. The study eye was bleached using a 0.25 ms flash at 8 × 10^4^ scot cd/m^2^s, equivalent to a 76% bleach, at a retinal location subtending 4 degrees and centered at 12 degrees inferiorly in the vertical meridian (location of the test target). The stimulus for the threshold measurement was a 2 degree diameter, 500 nm wavelength circular target presented for 200 ms which began 15 seconds after the bleaching offset. The initial stimulus presentation was at 1 log units of stimulus attenuation. Log thresholds were estimated using a modified, 3 down-1 up staircase procedure. The procedure continued with a 15 second break between each threshold measurement. This continued until either the rod-intercept time (RIT) was obtained, or the test protocol ended (30 minutes), whichever first occurred. RIT is defined as time taken for retinal sensitivity to recover to reach a threshold located within the second component of rod recovery (5 × 10^−3^ scot cd/m^2^ [3 log units of stimulus attenuation]). When the RIT was not obtained within the test duration, a capped value of 30 minutes was assigned for analysis. This procedure has been used previously to assess dark adaptation in cohorts with AMD.^21^^,^^22^

AdaptDx records the percentage of threshold points which indicate a fixation error. As in previous reports,^22^ if the fixation errors exceeded 30%, the test was deemed unreliable and excluded. Where fixation error rate was between 30% and 40%, or recovery occurred faster than 2 minutes, test data were evaluated manually by author Alison M. Binns (while masked to AMD status) to determine eligibility for inclusion according to specific criteria. Specifically, data were excluded where sensitivity showed a stepwise decrease over time indicative of the participant having been exposed to a light source during the dark adaptation process, or where a non-physiologically plausible jump in sensitivity recovery occurred (of greater than 2.0 log units between neighboring thresholds). Data were also excluded where sensitivity immediately post bleach was greater than 3.0 log units. Other reasons for exclusion of data were if the rod intercept could not be calculated by the device due to ineffective bleach delivery (e.g. due to fixation loss at the time of bleaching), or if the test was terminated early by the technician due to participant fatigue. When practical, participants repeated the test after a 30-minute washout period if their test data were deemed unreliable.

A scheme for data quality control and data export was followed as set out in the standard operating procedures (Supplement 1) and technicians had to pass a certification process (written test and submission of sample data) before they were approved to collect trial data. As reasoned before, we only considered data from participants who had successfully recorded BCVA at both visits. First, we identified missing data, following the standard operating procedure for what we describe as “examination procedural errors” (screening phase 1) excluding participants because of problems, for example, with the examination set-up (technician responsibility), even though they had BCVA recorded. Next, we identified missing data for “participant issues” (screening phase 2) resulting from, for example, abandoned examination, even though they had BCVA recorded. Finally, we identified data, following MACUSTAR protocols, deemed unreliable (screening phase 3) because of, for example, too many fixation errors, insufficient dark adaption, and incomplete bleaching. Results from this exercise alone will be useful for those planning future studies/trials wanting to estimate attrition rates of data when using measurements from S-MAIA and AdaptDx.

We used the main instrument determined summary measures of visual function as the measurements of interest, namely mesopic S-MAIA average (mean) threshold (MMAT decibels [dB]); scotopic S-MAIA average (mean) threshold (SMAT [dB]) and RIT (mins). Descriptive statistics were calculated for these measures along with age and BCVA at baseline for the different participant groups. For our primary analysis, test-retest variability for MMAT, SMAT, and RIT were estimated by the difference in the respective indices at baseline and the follow-up visit. Bland-Altman analysis was used to generate 95% limits of agreement calculated as ±1.96 times the standard deviation (SD) of the test-retest differences,^23^ with the mean of these differences, denoted bias, being an estimate of the average magnitude of practice or learning effect between sessions. The upper limits of agreement from the Bland-Altman analysis can be loosely interpreted as a value for the smallest detectable change that needs to be observed to be confident that the observed change is real and not a potential product of measurement error in the instrument.

For fairness of comparison between the two instrument types, RIT data were also transformed by 10 x log_10_ to mimic the logged (dB) output of the S-MAIA. A ratio of variability metric was calculated to also compare the test-retest variability performance of the measures, defined as the SD of the test-retest differences (noise) divided by SD of the average (signal), with the latter being the average of the measurement recorded over the two visits. A relatively large value for this metric would indicate large test-retest differences (high level of noise) and/or a small dynamic range (short span over the values for the averages). For completeness, we also calculated the intra-class correlation coefficient (ICC) for each measure using the one-way random-effects model.^24^ We restricted our primary analysis to participants defined as having iAMD.

To compare discrimination performance among the three measures to separate iAMD from early AMD, late AMD, and controls, receiver operating curves (ROC) and area under the curve (AUC) values with 95% confidence intervals (CIs) were computed for baseline data. Note age was not adjusted for in these analyses because we are only making relative comparisons between the measures. All data analyses were performed in R software version 4.0.5 (http://www.r-project.org/) under R Studio, version 1.1.463 (RStudio, Boston, MA, USA) including use of *ggplot2*, *BlandAltmanLeh*, *irr*, and *pROC* packages.

## Results

Three hundred one people participated in the MACUSTAR cross sectional study. Of these, 290 participants attended both visits and had complete BCVA data (controls [*n* = 54], early AMD [*n* = 28], iAMD [*n* = 167], and late AMD [*n* = 41]). See Table 1 for demographic and clinical details. Median time between sessions was 14 days (interquartile range [IQR] = 12–18 days). No disease progression between baseline and follow-up visits was observed.

**Table 1.** Baseline Demographic and Clinical Details of 290 Participants at Baseline

				Gender		Ethnicity			
Group	*N*	Mean Age in Years at Baseline (±SD)	Mean BCVA, logMAR (±SD)	Male	Female	Asian	African	Caucasian	Other
Controls	54	68 (6)	−0.04 (0.08)	22	32	0	0	54	0
Early AMD	28	72 (6)	0.01 (0.08)	6	22	0	0	28	0
iAMD	167	71 (8)	0.02 (0.10)	61	106	1	1	164	1
Late AMD	41	75 (6)	0.78 (0.24)	22	19	0	0	40	1

Demographic data were recorded via self-report.

BCVA, best corrected visual acuity; SD, standard deviation.

**Figure 1.**
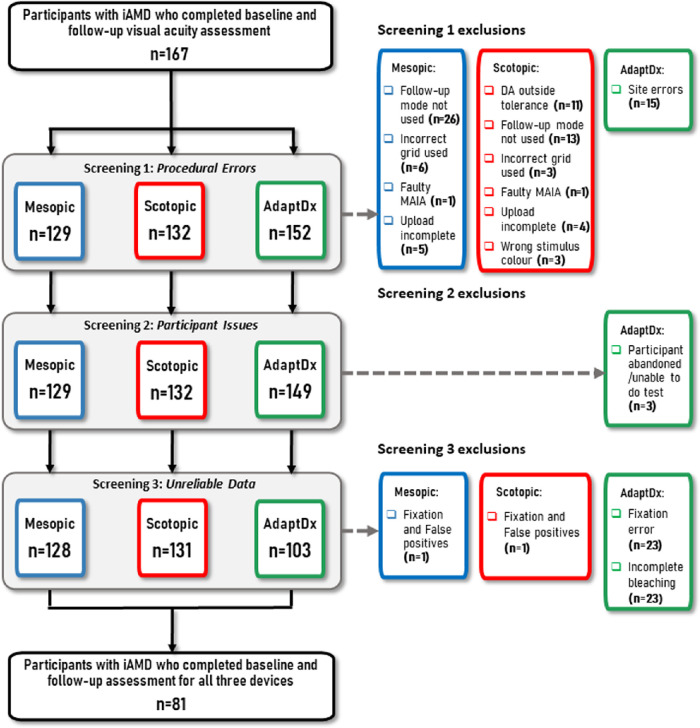
**Flowchart of participant screening with intermediate age-related macular degeneration (iAMD).** In some cases, multiple reasons for removal were recorded for a participant. In this case, whatever reason that occurred first in the screening processes was reported here. For example, if a participant was recorded as having “DA outside tolerance” (screening 1 exclusion) and recorded an unsuitable false-positive rate (screening 3 exclusion), the participant was removed based on the screening 1 exclusion. DA, dark adaptation.

Results from the data screening exercise for the participants with iAMD are shown in the flow-chart in Figure 1. A large quantity of data had to be excluded for mesopic (*n* = 39; 23%) and scotopic (*n* = 36; 22%) microperimetry with a higher attrition rate for AdaptDx data (*n* = 64; 38%). A large proportion of these data for all tests were excluded because of procedural errors (screening phase 1; *n* = 38 (23%) mesopic, *n* = 35 (21%) scotopic, and *n* = 15 (9%) AdaptDx, respectively). A proportion of data was excluded in screening phase 3 (unreliable data because of fixation errors and incomplete bleaching) for AdaptDx (*n* = 46; 27%). In short, we had complete data for 128, 131, and 103 participants for the mesopic and scotopic microperimetry data and AdaptDx data, respectively, and this was used in our primary analysis. We also grouped data for 81 (49%) of the 167 participants with iAMD who were able to complete all three tests. Results are presented in the same way for controls, early AMD, and late AMD in (Supplementary Figs. S1–S3). For example, for the 41 people with late AMD, a large proportion of RIT data was excluded (80%), with 76% of this due to unreliable data (due to fixation errors and incomplete bleaching).

**Figure 2.**
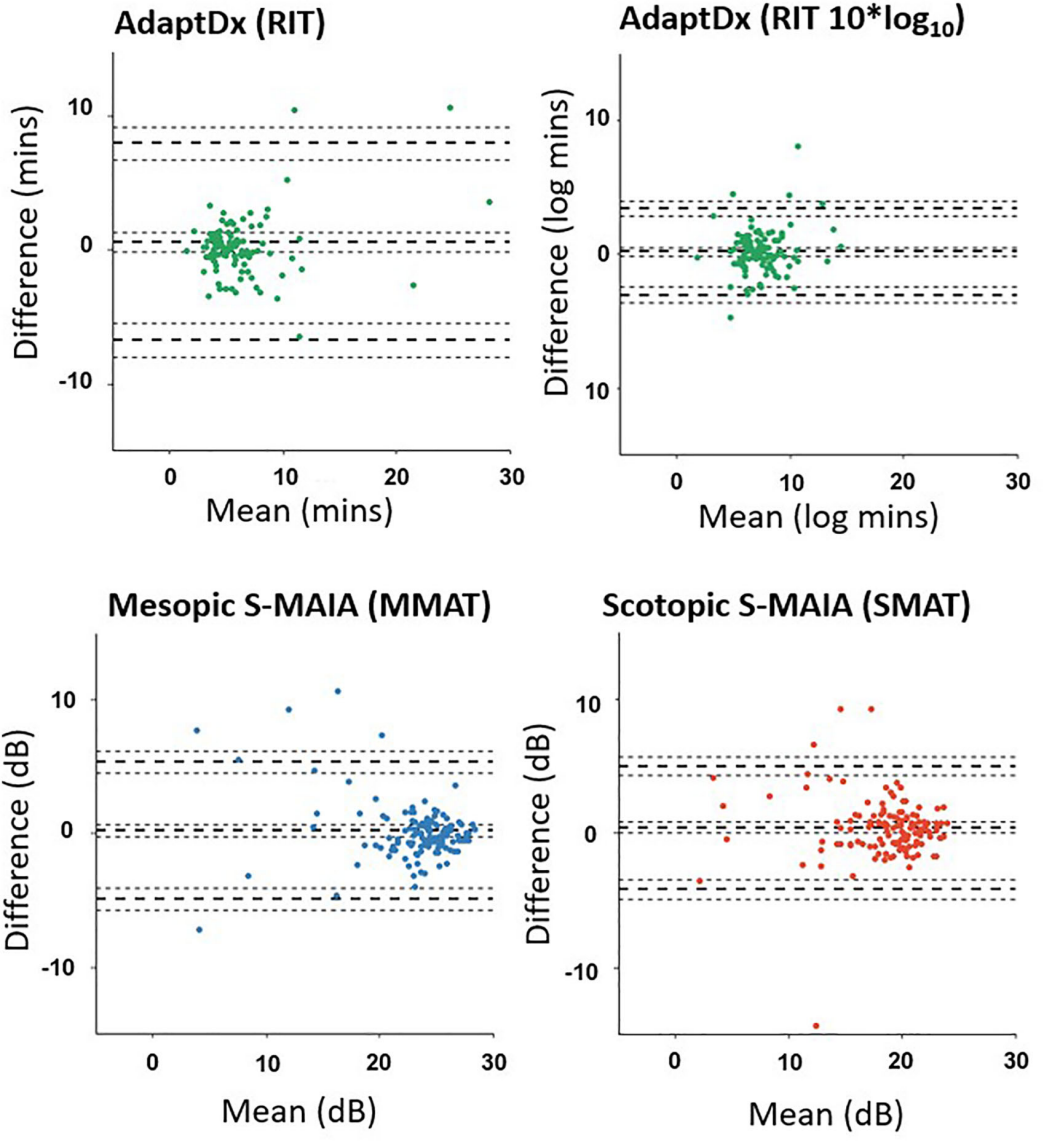
Bland-Altman plots to show the test-retest agreement for the three devices for participants with intermediate age-related macular degeneration (iAMD). Note: RIT data has been transformed by 10*log_10_ to mimic the logged output of the S-MAIA to allow for comparison of proportional changes, rather than measuring linear differences. Rod intercept time (RIT); mean mesopic average threshold (MMAT); mean scotopic average threshold (SMAT). dB, decibels.

Test-retest variability estimates for MMAT, SMAT, and RIT for the iAMD participants are described in Table 2 and Figure 2. MMAT and SMAT had very similar test-rest variability. The upper limits of agreement (smallest detectable change) were about 5 dB for both devices. It is difficult to compare Bland-Altman plots when the measures under scrutiny are recorded on different scales. Still, Bland-Altman plots for RIT (and the transformed RIT) seem similar to those for MMAT and SMAT. None of the Bland-Altman plots indicate heteroskedastic behavior. Moreover, the 95% CIs for bias in MMAT, SMAT, or RIT (see Table 2) indicate no evidence of better sensitivity or shorter RIT at the follow-up test compared to the baseline measurement; this suggests no improvement in performance between visits (practice/learning effect). Taken together, the different test-retest variability estimates for RIT (variability ratio and ICCs) were worse than those returned for MMAT and SMAT. For example, the ICC 95% CI for RIT (0.55, 0.77) did not overlap with the ICC 95% CI for MMAT (0.79, 0.89) and SMAT (0.78, 0.89; see Table 2). The upper limits of agreement (smallest detectable change) for RIT were about 8 minutes in the untransformed data.

**Table 2.** Test-Retest Variability Assessment Results for AdaptDX (Rod Intercept Time [Mins]) and S-MAIA (Mean Mesopic Average Threshold [dB] and Mean Scotopic Average Threshold [dB]) in People With Intermediate Age-Related Macular Degeneration (iAMD)

Test	*N*	Mean Baseline (±SD)	Mean Follow-Up (±SD)	Bias (95% CI)	SD of Differences	Lower Limits of Agreement (95% CI)	Upper Limits of Agreement (95% CI)	Interclass Correlation Coefficient (95% CI)	Variability Ratio
RIT (mins)	103	6.82	6.20	0.62	3.75	−6.73	7.96	0.67	0.88
		(5.41)	(3.76)	(−0.12, 1.34)		(−7.99, −5.47)	(6.70, 9.22)	(0.55, 0.77)	
RIT (10*log_10_)	103	7.60	7.40	0.20	1.65	−3.03	3.43	0.63	0.82
		(2.30)	(2.04)	(−0.12, 0.52)		(−3.58, −2.48)	(2.88, 3.98)	(0.49, 0.73)	
MMAT (dB)	128	23.12	22.89	0.23	2.62	−4.90	5.36	0.85	0.58
		(4.25)	(5.14)	(−0.23, 0.69)		(−5.69, −4.12)	(4.58, 6.15)	(0.79, 0.89)	
SMAT (dB)	131	18.68	18.27	0.42	2.35	−4.18	5.02	0.84	0.58
		(4.15)	(4.27)	(0.01, 0.82)		(−4.88, −3.49)	(4.32, 5.71)	(0.78, 0.89)	

dB, decibels; SD, standard deviation; 95% CI, 95% confidence interval.

We present secondary results from a similar analysis as applied to visually healthy controls, early AMD and late AMD. Test-retest variability estimates for MMAT, SMAT, and RIT for controls, early AMD and late AMD groups are given in Supplementary Figures S4–S6 and Supplementary Tables S2–S4. Note the smaller sample sizes for these groups which limits any comparison. For the small group of participants with early AMD the test-retest measures appeared to be better in RIT when compared to MMAT and SMAT but note, for example, the ICC 95% CI overlap for all three measures. Note that very few participants with late-stage AMD (*n* = 8) yielded RIT data as most was excluded in screening phase 3 (unreliable data because of fixation errors and incomplete bleaching). We also present results of the same measures stratified by individual study centers (*n* = ≥10 participants and iAMD only) to identify any cross-center effects in test-retest variability. This is an important observation, as for the first time, it has been confirmed that with these two devices “reliable” data can be collected in a multicenter trial setting. Data showing test-retest measures and participant screening/attrition stratified by individual study centers are given in Supplementary Table S1. These data show, for the most part, participants at different centers had consistent levels of moderate to excellent levels of reliability for all three devices (ICC = >0.5), with only two centers (CS0011 and CS015) indicating poorer levels (ICC = <0.5; for SMAT and RIT, respectively).

**Figure 3.**
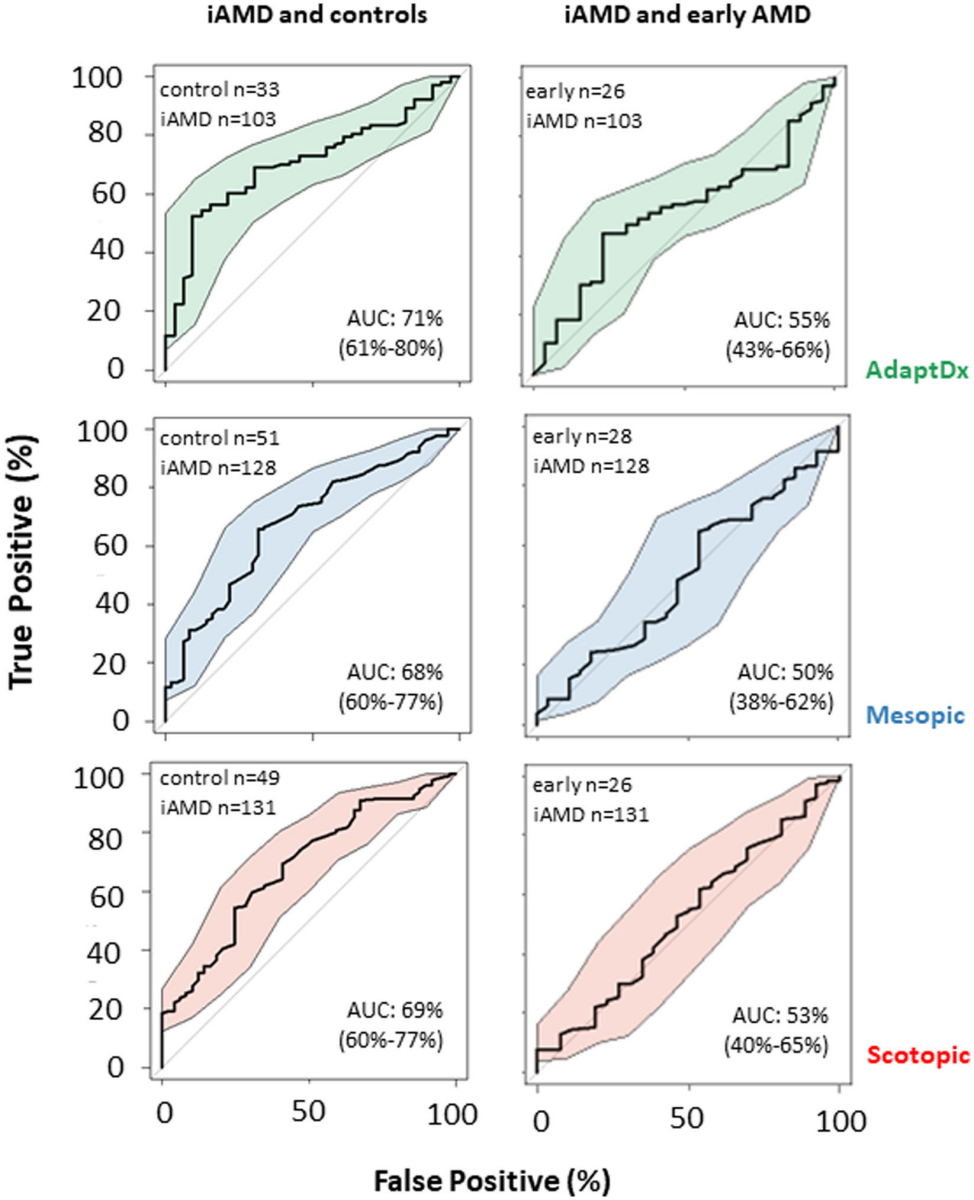
Receiver operating characteristic curves (ROC) comparing discrimination performance among the three measures’ individual ability to separate comparing healthy controls (no age-related macular degeneration [AMD]) and early stage AMD with intermediate AMD using baseline data. Area under the curve (AUC) and 95% CIs (shading) for each curve are provided.

The results of our discrimination analysis are summarized in Figure 3. MMAT, SMAT, and RIT had fair and equivalent discriminatory power when distinguishing between people with iAMD and controls. Yet, all three methods fail to distinguish between people with iAMD and early AMD.

We give AUC values for all contrasts among controls, early AMD, iAMD, and late AMD for the three different measures in Supplementary Tables S5–S7. Interpretation of these comparisons is hamstrung by small sample size for the non-iAMD groups. Yet, we think it interesting that AUC for contrasts between early AMD and controls were approximately 70% indicating fair discriminatory power for these measures when distinguishing between people with early AMD and controls.

**Figure 4.**
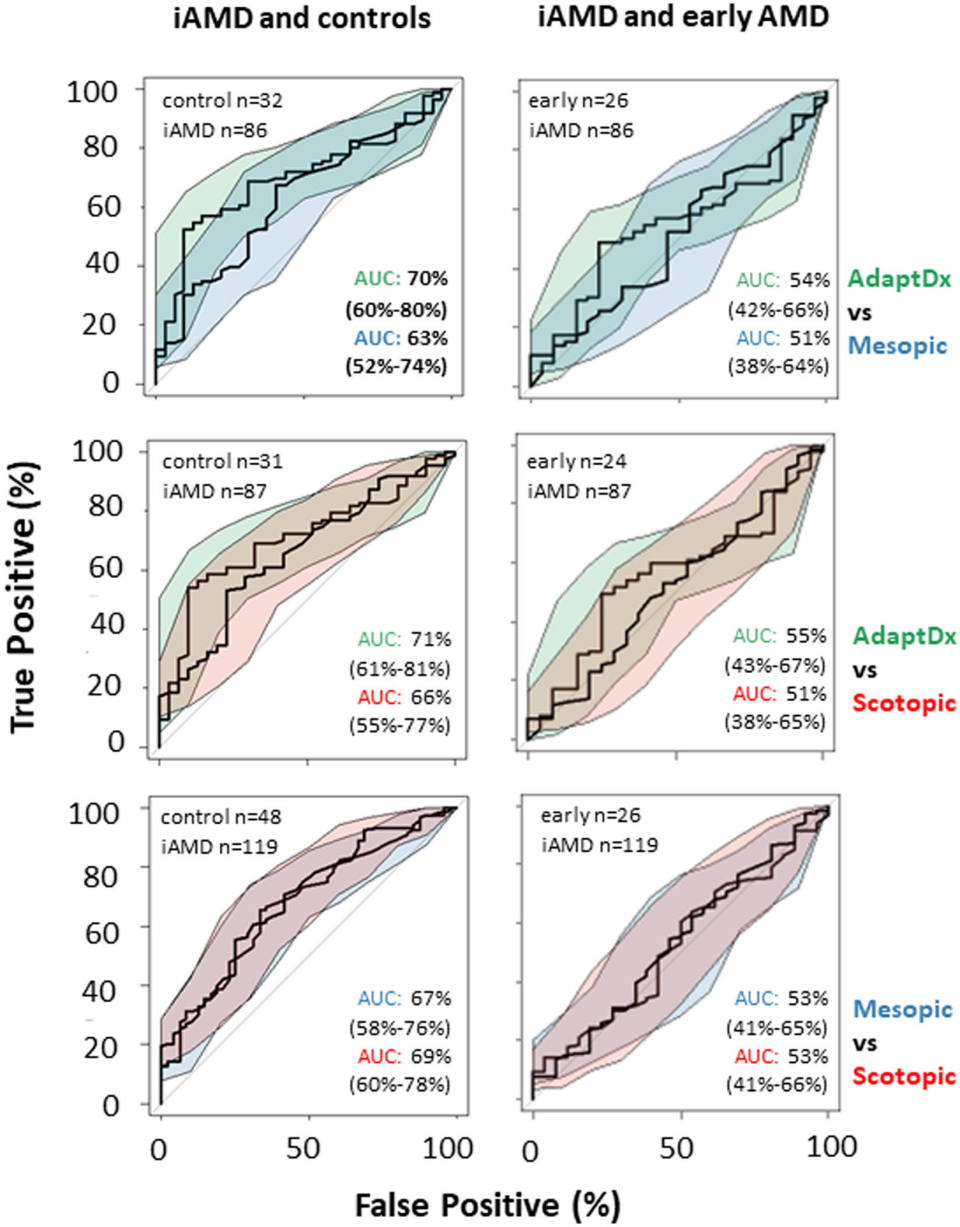
Receiver operating characteristic curves (ROC) comparing discrimination performance between the three measures’ individual ability to separate comparing healthy controls (no age-related macular degeneration [AMD]) and early stage AMD with intermediate AMD for the AdaptDx and S-MAIA, when used in tandem. Area under the curve (AUC) and 95% CIs (shading) for each curve are provided. (Note the smaller sample sizes representing participants from each group that performed both tests plotted e.g. 24 participants with early AMD successfully performed both AdaptDx and scotopic S-MAIA testing.)

In order to compare all three measurements head-to-head for their discriminatory power, a different comparison using data where a participant had successfully recorded all three measurements yielded similar results with MMAT, SMAT, and RIT having equivalent discriminatory power distinguishing between iAMD subjects and controls (see Fig. 4, Supplementary Table S8).

## Discussion

We assessed mesopic (MMAT) and scotopic (SMAT) microperimetry and dark adaptation (RIT) in a large number of people with iAMD from the multicenter MACUSTAR cross-sectional study. A proportion of test results from these devices could not be used despite assessments being performed following consistent standard operating procedures. Measurements from these devices had similar levels of test-retest variability with some lines of evidence that it was worse with RIT (see ICC values in Table 2, for example). In a secondary analysis, we found MMAT, SMAT, and RIT to be equivalent in having fair discriminatory power when separating iAMD subjects and controls (and early AMD versus controls). Yet, all three assessments were unable to discriminate between early AMD and iAMD subjects. Baseline and follow-up measurements in these device-based tests were similar, indicating no evidence of learning or practice effects; this is surprising given what is often experienced in study participants in perimetry and other psychophysical measures of visual function.^25^^,^^26^ Our standard operating procedures will be useful for future trials using device-based assessments, including short practice tests, mitigating any learning effect risk. When followed, the standard operating procedures work in a multicenter setting and aid the generation of interpretable and reliable data (available in Supplementary 1).

Results from our study are informative for future iAMD clinical trials using S-MAIA and AdaptDx. Having good estimates of test-retest variability from these instrument's metrics and estimates of usable/reliable data are invaluable for trial design and sample size calculations. Our results highlight challenges with these device-based measures of visual function. For example, data from a large number of people who could successfully perform a chart-based visual function test (BCVA) had to be excluded for mesopic and scotopic microperimetry, mainly due to procedural errors (21% and 23%, respectively), despite the use of standard operating procedures. This attrition rate was worse for AdaptDx data (38%). However, patient fatigue is likely a major issue as the device-based testing was performed at the end of the visit after completing a battery of other tests and the dark adaptation assessment alone lasted up to 30 minutes. Furthermore, this data attrition was worst in sites that collected data from <10 people, highlighting the likely need for experienced assessors at the clinical sites less familiar with more recent devices. SMAT also varied a lot between sites (see Supplementary Table S1), suggesting MMAT would be preferable in a multicenter setting. Interestingly, ICC values for MMAT and SMAT are no worse than those reported in our companion study of simpler to administer chart-based tests.^9^ Furthermore, data we report here cannot predict the potential of these more complicated test modalities in tracking disease progression, which is being assessed currently in longitudinal component of MACUSTAR. Indeed, other recently reported longitudinal studies, albeit on a smaller scale, have already demonstrated the promise of microperimetry and adaptometry in detecting visual function changes resulting from progression of iAMD in relatively short follow-ups.^27^^,^^28^

Some more discussion of our secondary analyses, in what we describe as discrimination performance of the S-MAIA and AdaptDx, is pertinent. First, the MACUSTAR cross-sectional study was not designed to truly assess the diagnostic accuracy of these device-based tests. None of these analyses were powered appropriately because the sample sizes for the non iAMD groups were small and what we report would not satisfy guidelines for diagnostic accuracy studies.^29^ Furthermore, our AUCs are constructed on a smaller data set due to more missing data meaning those estimates are biased with the likelihood that discrimination is worse than estimated here. After all, missing data are often not random but results from a test not being completed because of, for example, unreliability or lack of participant compliance. (As a statistical aside, this uncertainty in the estimates is not reflected by greatly wider CIs for the AUC.)^30^ Nevertheless, the present evidence suggests that MMAT, SMAT, and RIT have equivalent discriminatory power when distinguishing between iAMD subjects and controls, for example. AUC values we report were equivalent to the measurements from most of the chart-based tests reported in our companion paper but not as good as contrast sensitivity measured by Pelli-Robson, which afforded best discrimination between iAMD and controls (AUC = 0.77)^9^; this is noteworthy given the convenience of chart-based tests. At the same time, the device-based tests of visual function seemed to have better AUCs than the chart based studies when separating controls from subjects with early AMD, but the sample size of these groups limit interpretability of these results (see Supplementary 1; Supplementary Tables S5–S7). Of course, we do not know how any of these measures will perform in detecting disease progression and this is the subject of longitudinal component of MACUSTAR.

Interestingly, measurements from scotopic and mesopic microperimetry have a very similar profile. MMAT and SMAT offer almost identical test-retest variability and have very similar discriminatory power too. The latter suggest that mesopic microperimetry ought to be a first choice because it is a more convenient examination to do. Yet, we do not know how measurements from these two modalities will perform in the longitudinal data from MACUSTAR. It might be that scotopic microperimetry may pick up subtle changes in iAMD subjects that might be predictive of progression to advanced disease.^31^ Presently, the more inconvenient test, and perhaps challenging test for the patient, does not yield a great level of measurement noise (worse test-retest variability); this is useful new knowledge supporting findings from smaller studies.^32^

There are few reports in the literature similar to ours. Flamendorf et al. (2015) assessed test-retest variability between RIT measured in 2 visits (follow-up mean = 7 days) in 87 people with AMD using the AdaptDx. RIT mean (± SD) difference was 0.02 (± 2.26) minutes.^11^ AMD severity of these participants was not reported, but they were likely “functionally normal” or early stage AMD as they did not reach the AdaptDx test ceiling^8^^,^^11^ and this is notable. Hess et al. (2022) assessed test-retest variability of the RIT using the AdaptDX. Analysis was done on data from tests conducted approximately 2 weeks apart in 191 people with varying levels of AMD and no AMD. Their measurement of RIT had a value of ICC = 0.88 which was considerably higher than what we report in the present study. Hess et al. suggested RIT to be a suitable outcome measure to be used in clinical trials.^33^ Test–retest variability of RIT has also been assessed using other instruments. For example, Uddin et al. (2020) evaluated test-retest variability of measures of RIT from the Medmont DAC (MDAC) perimeter but this was done in a very small sample (*n* = 9 with iAMD and *n* = 3 controls). The authors reported the coefficient of repeatability (CoR) of RIT data was 7.6 minutes without any limits of precision around their estimate.^34^

Elsewhere in the literature, there is overwhelming evidence of an association between impaired dark adaptation and AMD but the studies of the discrimination performance of RIT in separating people with different levels of AMD are limited.^10^ Good levels of test-retest variability of the S-MAIA (both mesopic and scotopic conditions) have been previously reported using CoR metrics. For example, Welker et al. (2018) reported CoR of 4.4 dB (mesopic) and 4.52 dB (scotopic) for pointwise sensitivity in a small number (*n* = 23) of volunteers with iAMD.^12^ Barkana et al. (2021) reported test-retest variability of “abnormal” microperimetry points only (defined if threshold sensitivity was at least 5% lower than expected values in healthy eyes).^35^ Pfau et al. (2017) assessed 47 visually healthy eyes and reported slightly worse CoRs than Welker et al. (2018; 4.75 dB and 4.06 dB, respectively).^13^ Similarly, under mesopic conditions using the unmodified MAIA with a 37 point grid, Wu et al. (2013) compared controls and people with iAMD and found CoR of 4.12 and 3.74 dB, respectively.^36^ von der Emde et al. (2019) assessed test-retest variability in 28 people with neovascular AMD in both cyan and red scotopic testing conditions using the S-MAIA and reported CoR of 6.14 dB and 6.06 dB, respectively.^37^ CoR has also been assessed in other microperimeters. For example, Grewal et al. (2021) compared test-retest variability for scotopic pointwise sensitivity with MDAC perimeter. CoR was 5.96 dB and 5.09 dB (cyan and red, respectively).^38^ We used ICC and a variability ratio metric in our work. With the latter, measurements from the devices are penalized both for having large test-retest variability (large noise) and for having small dynamic range (short span over the values for the averages).

Few studies in the literature have assessed discriminatory performance of the S-MAIA in people with AMD. Pondorfer et al. (2020) reported that the S-MAIA could successfully discriminate among 83 people with iAMD and 24 controls in a study performed at one center. AUC values were better than what we reported (88% mesopic and 82% scotopic). Still, CIs around these estimates were wider reflecting the smaller sample sizes.^39^ Interestingly, their study showed Pelli-Robson contrast sensitivity to have the highest AUC value (95% CI of 0.81, 0.97) when compared to other visual function tests they considered; and this supports our other findings on chart based tests.^9^

Our study has strengths and limitations. The data set is unique because of its size, being yielded from a multicenter setting, and following freely available standard operating procedures. We did not randomize the order of the visual function assessments in the MACUSTAR cross-sectional study and this is a limitation. In addition, the use of a single microperimetry and dark adaptation device might be considered a limitation of our study. Yet, the inclusion of more devices would have not been practically feasible for the MACUSTAR study. Indeed, the device-based testing reported here was done at the end of series of chart-based tests and other assessments. As stated before, this might explain some of the data attrition especially with the dark adaptation assessment scheduled at the very end of an extensive examination session. There are minor limitations associated with what we describe as our discriminatory analyses, such as not correcting estimates of AUC for age, sex, or phakic status but we have already outlined that these analyses were comparative and never designed as a formal assessment of diagnostic accuracy of the device-based tests.

## Conclusion

To sum up, we have reported on the properties of measurements from device-based testing of visual function, namely mesopic and scotopic microperimetry and dark adaptation. The standard operating procedures, estimates of test-retest variability and test completion rates will inform the design of future AMD trials. The results from the longitudinal component of MACUSTAR will inform further on the prognostic power of measurements from these three devices and on their capability to track for iAMD progression.

## Supplementary Material

Supplement 1

## Appendix

MACUSTAR consortium members: H. Agostini, L. Altay, R. Atia, F. Bandello, P. G. Basile, J. Batuca, C. Behning, M. Belmouhand, M. Berger, A. Binns, C. J. F. Boon, M. Böttger, C. Bouchet, J. E. Brazier, T. Butt, C. Carapezzi, J. Carlton, A. Carneiro, A. Charil, R. Coimbra, M. Cozzi, D. P. Crabb, J. Cunha-Vaz, C. Dahlke, L. de Sisternes, H. Dunbar, R. P. Finger, E. Fletcher, H. Floyd, C. Francisco, M. Gutfleisch, S. Hinz, R. Hogg, F. G. Holz, C. B. Hoyng, A. Kilani, J. Krätzschmar, L. Kühlewein, M. Larsen, S. Leal, Y. T. E. Lechanteur, U. F. O. Luhmann, A. Lüning, I. Marques, C. Martinho, G. Montesano, Z. Mulyukov, M. Paques, B. Parodi, M. Parravano, S. Penas, T. Peters, T. Peto, M. Pfau, S. Poor, S. Priglinger, D. Rowen, G. S. Rubin, J. Sahel, C. Sánchez, O. Sander, M. Saßmannshausen, M. Schmid, S. Schmitz-Valckenberg, J. Siedlecki, R. Silva, A. Skelly, E. Souied, G. Staurenghi, L. Stöhr, J. Tavares, D. J. Taylor, J. H. Terheyden, S. Thiele, A. Tufail, M. Varano, L. Vieweg, L. Wintergerst, A. Wolf, and N. Zakaria.

## References

[1] Finger RP, Schmitz-Valckenberg S, Schmid M, et al. MACUSTAR: development and clinical validation of functional, structural, and patient-reported endpoints in intermediate age-related macular degeneration. *Ophthalmologica*. 2019; 241(2): 61–72. 30153664 10.1159/000491402 PMC6482983

[2] Saßmannshausen M, Steinberg JS, Fimmers R, et al. Structure-function analysis in patients with intermediate age-related macular degeneration. *Invest Ophthalmol Vis Sci*. 2018; 59(3): 1599–1608. 29625486 10.1167/iovs.17-22712

[3] Wong EN, Chew AL, Morgan WH, Patel PJ, Chen FK. The use of microperimetry to detect functional progression in non-neovascular age-related macular degeneration: a systematic review. *Asia-Pacific J Ophthalmol*. 2017; 6(1): 70–79. 10.22608/APO.201643 28161925

[4] Cassels NK, Wild JM, Margrain TH, Chong V, Acton JH. The use of microperimetry in assessing visual function in age-related macular degeneration. *Surv Ophthalmol*. 2018; 63(1): 40–55. 28579549 10.1016/j.survophthal.2017.05.007

[5] Yang Y, Dunbar H. Clinical perspectives and trends: microperimetry as a trial endpoint in retinal disease. *Ophthalmologica*. 2021; 244(5): 418–450. 33567434 10.1159/000515148 PMC8686703

[6] Owsley C, Huisingh C, Jackson GR, et al. Associations between abnormal rod-mediated dark adaptation and health and functioning in older adults with normal macular health. *Invest Ophthalmol Vis Sci*. 2014; 55(8): 4776–4789. 24854857 10.1167/iovs.14-14502 PMC4122017

[7] Owsley C, McGwin G, Clark ME, et al. Delayed rod-mediated dark adaptation is a functional biomarker for incident early age-related macular degeneration. *Ophthalmology*. 2016; 123(2): 344–351. 26522707 10.1016/j.ophtha.2015.09.041 PMC4724453

[8] Chen KG, Alvarez JA, Yazdanie M, et al. Longitudinal study of dark adaptation as a functional outcome measure for age-related macular degeneration. *Ophthalmology*. 2019; 126(6): 856–865. 30278196 10.1016/j.ophtha.2018.09.039 PMC8380039

[9] Dunbar HMP, Behning C, Abdirahman A, et al. Repeatability and discriminatory power of chart-based visual function tests in individuals with age-related macular degeneration: A MACUSTAR study report. *JAMA Ophthalmol*. Published online June 23, 2022, 10.1001/JAMAOPHTHALMOL.2022.2113. PMC9227684 35737401

[10] Higgins BE, Taylor DJ, Binns AM, Crabb DP. Are current methods of measuring dark adaptation effective in detecting the onset and progression of age-related macular degeneration? A systematic literature review. *Ophthalmol Ther*. 2021; 10(1): 21–38. 33565038 10.1007/s40123-020-00323-0 PMC7887145

[11] Flamendorf J, Agrón E, Wong WT, et al. Impairments in dark adaptation are associated with age-related macular degeneration severity and reticular pseudodrusen. *Ophthalmology*. 2015; 122(10): 2053–2062. 26253372 10.1016/j.ophtha.2015.06.023 PMC4836058

[12] Welker SG, Pfau M, Heinemann M, Schmitz-Valckenberg S, Holz FG, Finger RP. Retest reliability of mesopic and dark-adapted microperimetry in patients with intermediate age-related macular degeneration and age-matched controls. *Invest Ophthalmol Vis Sci*. 2018; 59(4): AMD152–AMD159. 30372731 10.1167/iovs.18-23878

[13] Pfau M, Lindner M, Fleckenstein M, et al. Test-retest reliability of scotopic and mesopic fundus-controlled perimetry using a modified MAIA (macular integrity assessment) in normal eyes. *Ophthalmologica*. 2017; 237(1): 42–54. 27997924 10.1159/000453079

[14] Terheyden JH, Holz FG, Schmitz-Valckenberg S, et al. Clinical study protocol for a low-interventional study in intermediate age-related macular degeneration developing novel clinical endpoints for interventional clinical trials with a regulatory and patient access intention - MACUSTAR. *Trials*. 2020; 21(1): 1–11. 32682441 10.1186/s13063-020-04595-6 PMC7368769

[15] Ferris FL, Wilkinson CP, Bird A, et al. Clinical classification of age-related macular degeneration. *Ophthalmology*. 2013; 120(4): 844–851. 23332590 10.1016/j.ophtha.2012.10.036 PMC11551519

[16] Saßmannshausen M, Thiele S, Behning C, et al. Intersession repeatability of structural biomarkers in early and intermediate age-related macular degeneration: A MACUSTAR study report. *Transl Vis Sci Technol*. 2022; 11(3): 27–27. 10.1167/tvst.11.3.27 PMC8963672 35333287

[17] Terheyden JH, Behning C, Lüning A, et al. Challenges, facilitators and barriers to screening study participants in early disease stages-experience from the MACUSTAR study. *BMC Med Res Methodol*. 2021; 21(1): 54. 33731014 10.1186/s12874-021-01243-8 PMC7967977

[18] Steinberg JS, Saßmannshausen M, Pfau M, et al. Evaluation of two systems for fundus-controlled scotopic and mesopic perimetry in eye with age-related macular degeneration. *Transl Vis Sci Technol*. 2017; 6(4): 7. 10.1167/tvst.6.4.7 PMC5509379 28713647

[19] Curcio CA, Medeiros NE, Millican CL. Photoreceptor loss in age-related macular degeneration. *Invest Ophthalmol Vis Sci*. 1996; 37(7): 1236–1249. 8641827

[20] Nebbioso M, Barbato A, Pescosolido N. Scotopic microperimetry in the early diagnosis of age-related macular degeneration: preliminary study. Mizota A, ed. *Biomed Res Int*. 2014; 2014: 671529. 25548774 10.1155/2014/671529 PMC4274674

[21] Binns AM, Taylor DJ, Edwards LA, et al. Determining optimal test parameters for assessing dark adaptation in people with intermediate age-related macular degeneration. *Invest Ophthalmol Vis Sci*. 2018; 59(4 PG-114-121): AMD114–AMD121. 30105357 10.1167/iovs.18-24211

[22] Jackson GR, Scott IU, Kim IK, Quillen DA, Iannaccone A, Edwards JG. Diagnostic sensitivity and specificity of dark adaptometry for detection of age-related macular degeneration. *Invest Opthalmol Vis Sci*. 2014; 55(3): 1427. 10.1167/iovs.13-13745 PMC3954002 24550363

[23] Martin Bland J, Altman DG. Statistical methods for assessing agreement between two methods of clinical measurement. *The Lancet*. 1986; 327(8476): 307–310. 2868172

[24] Koo TK, Li MY. A guideline of selecting and reporting intraclass correlation coefficients for reliability research. *J Chiropr Med*. 2016; 15(2): 155–163. 27330520 10.1016/j.jcm.2016.02.012 PMC4913118

[25] Jones PR, Yasoubi N, Nardini M, Rubin GS. Feasibility of macular integrity assessment (MAIA) microperimetry in children: sensitivity, reliability, and fixation stability in healthy observers. *Invest Ophthalmol Vis Sci*. 2016; 57(14): 6349–6359. 27898980 10.1167/iovs.16-20037

[26] Wu Z, Ayton LN, Guymer RH, Luu CD. Intrasession test-retest variability of microperimetry in age-related macular degeneration. *Invest Ophthalmol Vis Sci*. 2013; 54(12): 7378–7385. 24135753 10.1167/iovs.13-12617

[27] Lad EM, Fang V, Tessier M, et al. Longitudinal evaluation of visual function impairments in early and intermediate age-related macular degeneration patients. *Ophthalmology Science*. 2022; 2(3): 100173. 36245764 10.1016/j.xops.2022.100173 PMC9559970

[28] Hsu ST, Thompson AC, Stinnett SS, et al. Longitudinal study of visual function in dry age-related macular degeneration at 12 months. *Ophthalmol Retina*. 2019; 3(8): 637–648. 31060977 10.1016/j.oret.2019.03.010 PMC6684849

[29] Fidalgo BMRR, Crabb DP, Lawrenson JG. Methodology and reporting of diagnostic accuracy studies of automated perimetry in glaucoma: evaluation using a standardised approach. *Ophthalmic and Physiological Optics*. 2015; 35: 315–323. 25913874 10.1111/opo.12208

[30] Cho H, Matthews GJ, Harel O. Confidence intervals for the area under the receiver operating characteristic curve in the presence of ignorable missing data. *Int Stat Rev*. 2019; 87(1): 152–177. 31007356 10.1111/insr.12277 PMC6472951

[31] Pfau M, Lindner M, Gliem M, et al. Mesopic and dark-adapted two-color fundus-controlled perimetry in patients with cuticular, reticular, and soft drusen. *Eye (Lond)*. 2018; 32(12): 1819–1830. 30068928 10.1038/s41433-018-0183-3 PMC6292882

[32] Pfau M, Lindner M, Müller PL, et al. Effective dynamic range and retest reliability of dark-adapted two-color fundus-controlled perimetry in patients with macular diseases. *Invest Ophthalmol Vis Sci*. 2017; 58(6): BIO158–BIO167. 28692722 10.1167/iovs.17-21454

[33] Hess K, de Silva T, Grisso P, et al. Evaluation of cone- and rod-mediated parameters in dark adaptation testing as outcome measures in age-related macular degeneration. *Ophthalmol Retina*. 2022; 6(12). 10.1016/j.oret.2022.05.018 35643387

[34] Uddin DY, Jeffrey B, Wong W, et al. Repeatability and comparison of dark adaptation using the Medmont DAC perimeter and AdaptDx dark adaptometer. *Invest Ophthalmol Vis Sci*. 2019; 60(9).

[35] Barkana Y, Pondorfer SG, Schmitz-Valckenberg S, Russ H, Finger RP. Improved sensitivity of microperimetric outcomes for clinical studies in age-related macular degeneration. *Sci Rep*. 2021; 11(1): 4764. 33637858 10.1038/s41598-021-83716-w PMC7910434

[36] Wu Z, Ayton LN, Guymer RH, Luu CD. Intrasession test-retest variability of microperimetry in age-related macular degeneration. *Invest Ophthalmol Vis Sci*. 2013; 54(12): 7378–7385. 24135753 10.1167/iovs.13-12617

[37] von der Emde L, Pfau M, Thiele S, et al. Mesopic and dark-adapted two-color fundus-controlled perimetry in choroidal neovascularization secondary to age-related macular degeneration. *Transl Vis Sci Technol*. 2018; 8(1): 7. 30637177 10.1167/tvst.8.1.7 PMC6327348

[38] Grewal MK, Chandra S, Bird A, Jeffery G, Sivaprasad S. Scotopic thresholds on dark-adapted chromatic perimetry in healthy aging and age-related macular degeneration. *Sci Rep*. 2021; 11(1): 1–10. 33990634 10.1038/s41598-021-89677-4 PMC8121851

[39] Pondorfer SG, Heinemann M, Wintergerst MWM, et al. Detecting vision loss in intermediate age related macular degeneration: a comparison of visual function tests. *PLoS One*. 2020; 15(4). 10.1371/journal.pone.0231748 PMC7162506 32298375

