# Supplementary material for: Test-Retest Variability and Discriminatory Power of Measurements From Microperimetry and Dark Adaptation Assessment in People With Intermediate Age-Related Macular Degeneration – A MACUSTAR Study Report
Source: Transl Vis Sci Technol. 2023 Jul 21;12(7):19. doi: 10.1167/tvst.12.7.19 (PMC10365139; doi:10.1167/tvst.12.7.19)
Supplement: Supplement 1 [file tvst-12-7-19_s001.pdf]

Supplemental Online Content

Supplemental Figures

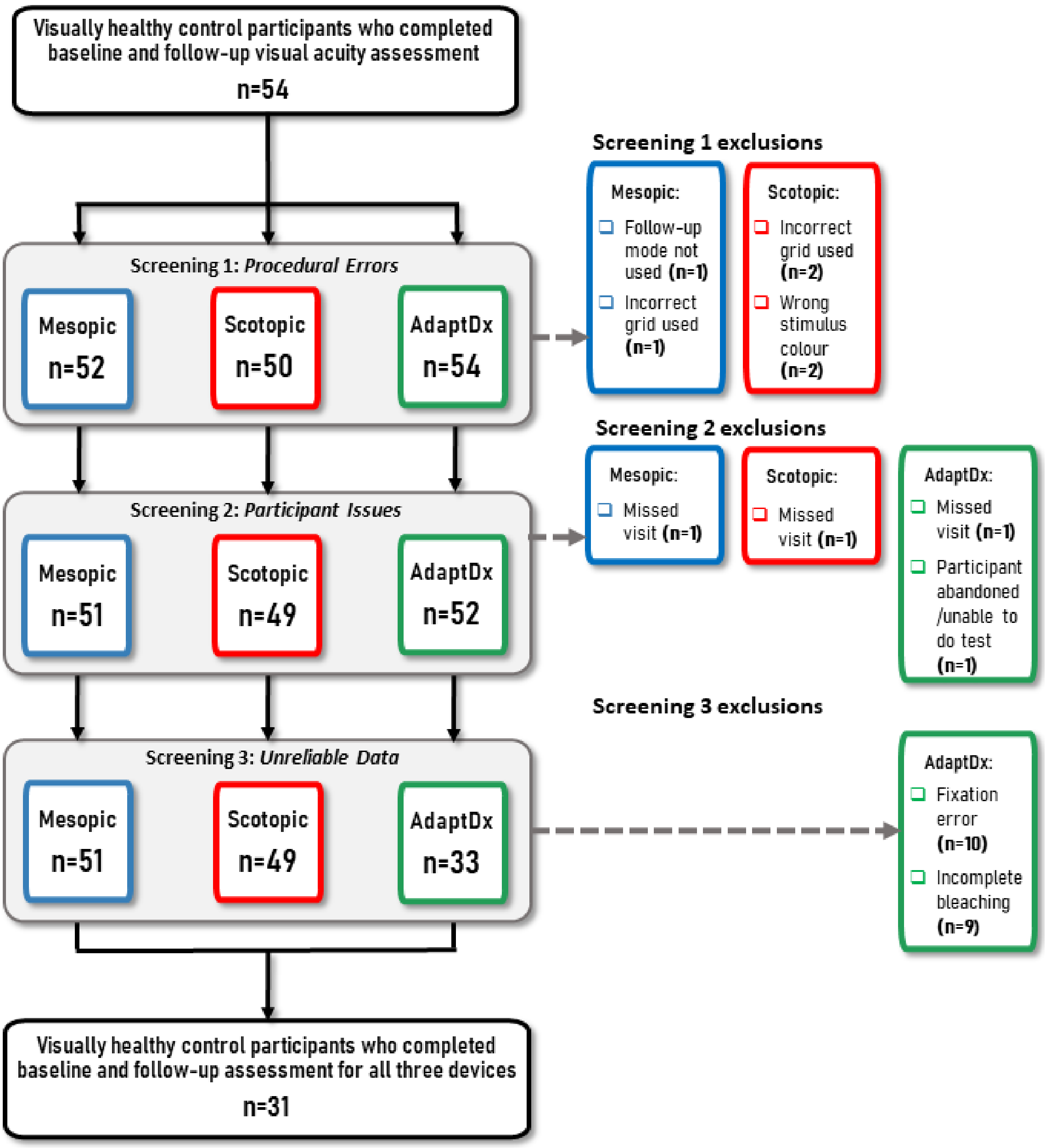

Figure S1. Flowchart Of Health Control Participant Screening. During Screening Phase 1, the datasets corresponding to the three methods used in this study were assessed for procedural errors. During Screening Phase 2, data was screened out if participant errors occurred e.g. participant abandoned

the test. Finally, for Screening Phase 3, the datasets were screened for unreliable data, deemed so by MACUSTAR protocol.

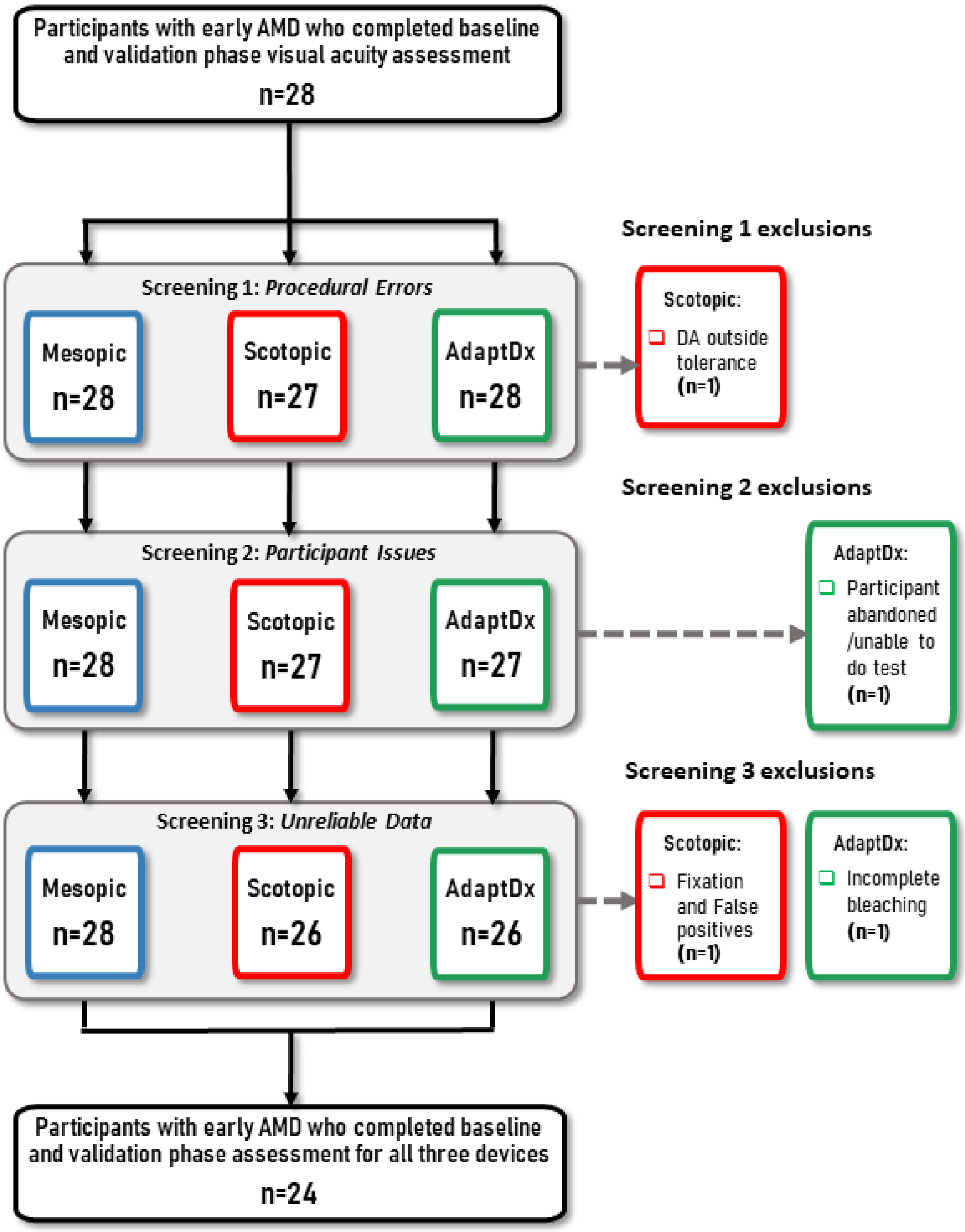

Figure S2. Flowchart Of Participant Screening With Early Age-Related Macular Degeneration (AMD). During Screening Phase 1, the datasets corresponding to the three methods used in this study were assessed for procedural errors. During Screening Phase 2, data was screened out if participant errors occurred e.g. participant abandoned the test. Finally, for Screening Phase 3, the datasets were screened for unreliable data, deemed so by MACUSTAR protocol.

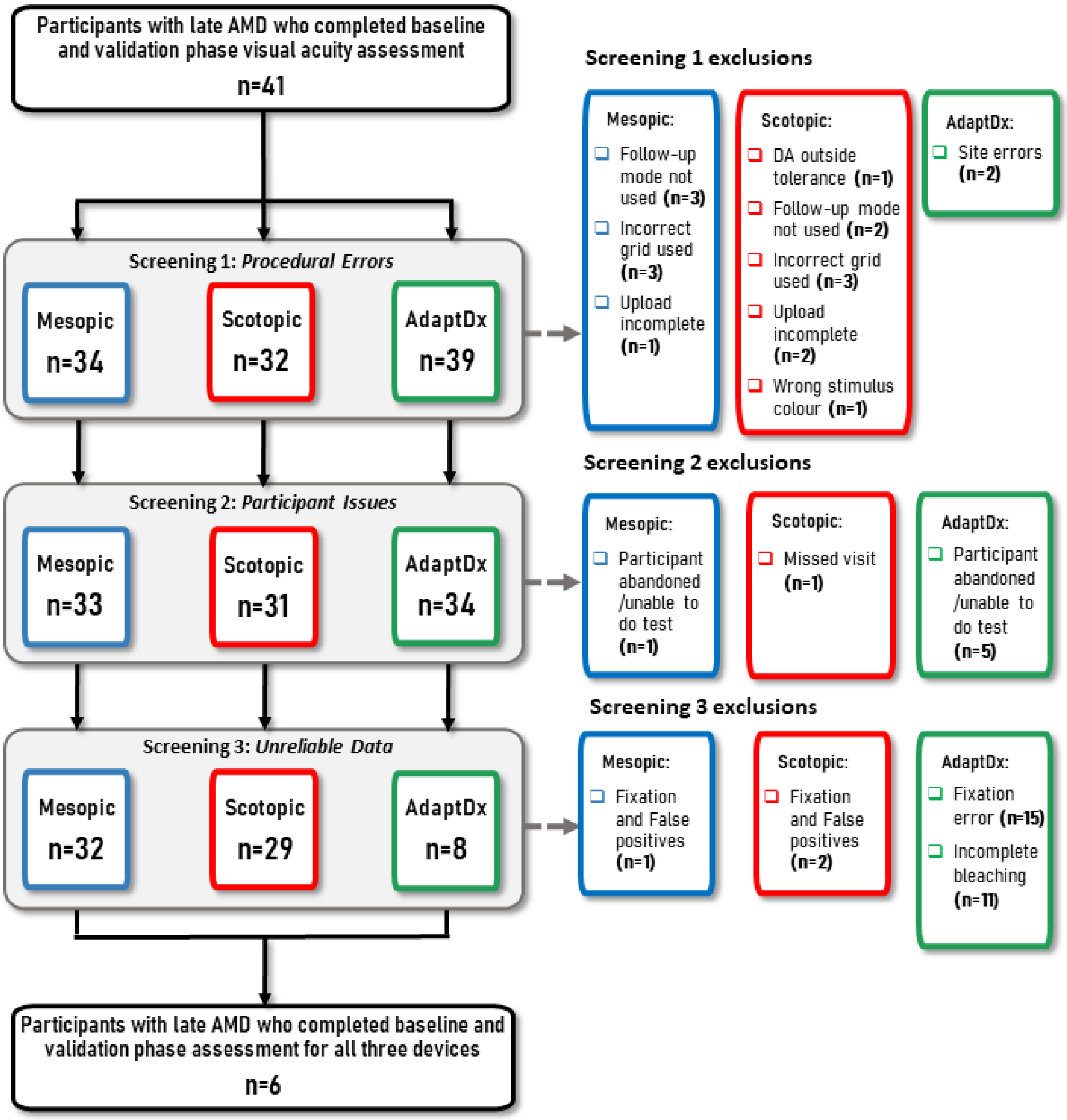

Figure S3. Flowchart Of Participant Screening With Late Age-Related Macular Degeneration (AMD). During Screening Phase 1, the datasets corresponding to the three methods used in this study were assessed for procedural errors. During Screening Phase 2, data was screened out if participant errors occurred e.g. participant abandoned the test. Finally, for Screening Phase 3, the datasets were screened for unreliable data, deemed so by MACUSTAR protocol.

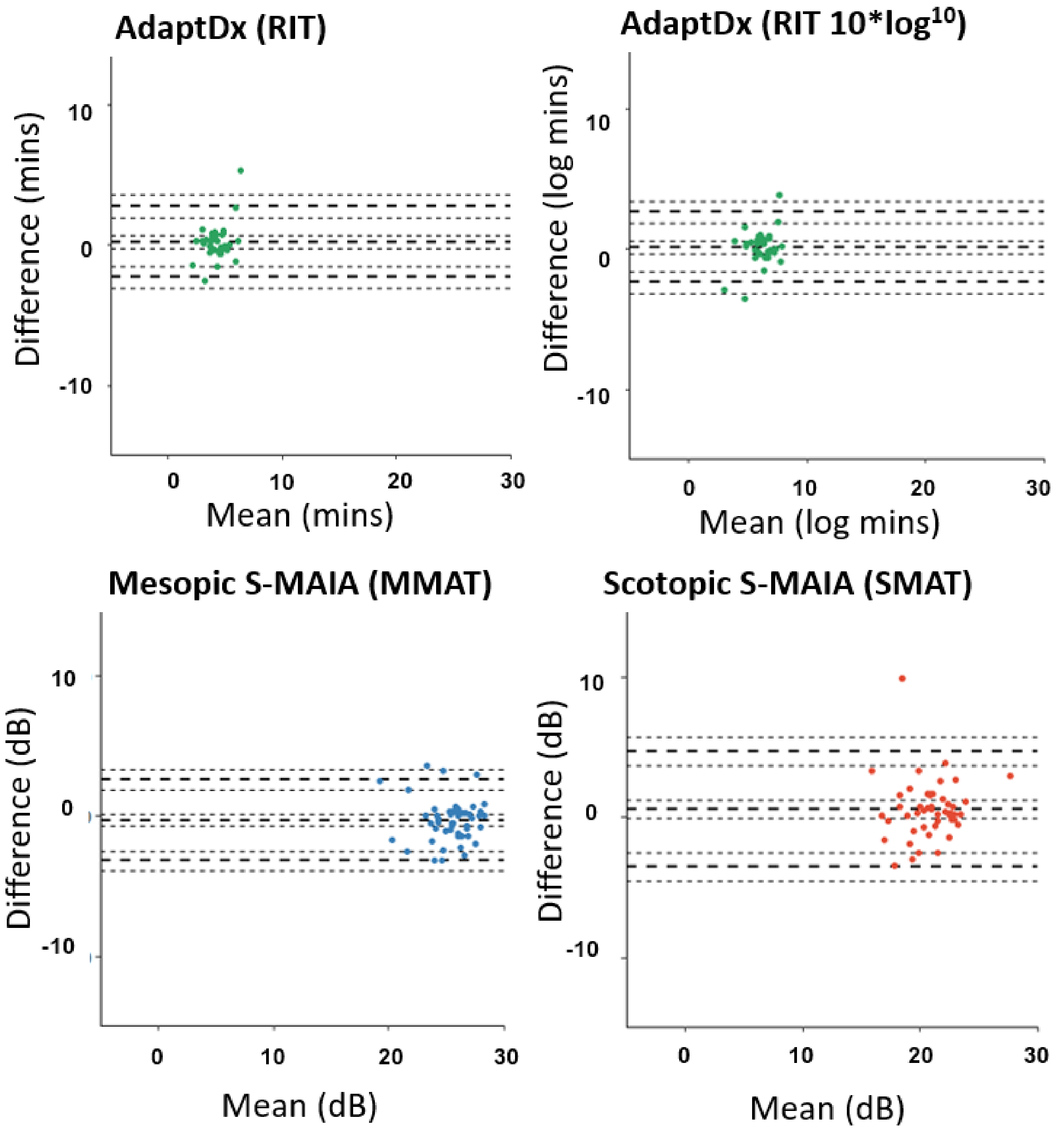

Figure S4. Bland-Altman Plots To Show The Test-Retest Agreement For The Three Devices For Control Participants). Note: Rod intercept time (RIT) has been transformed by 10*log10 to mimic the logged output of the S-MAIA for better comparison (top-right). Mean mesopic average threshold (MMAT); mean scotopic average threshold (SMAT); Decibels (dB).

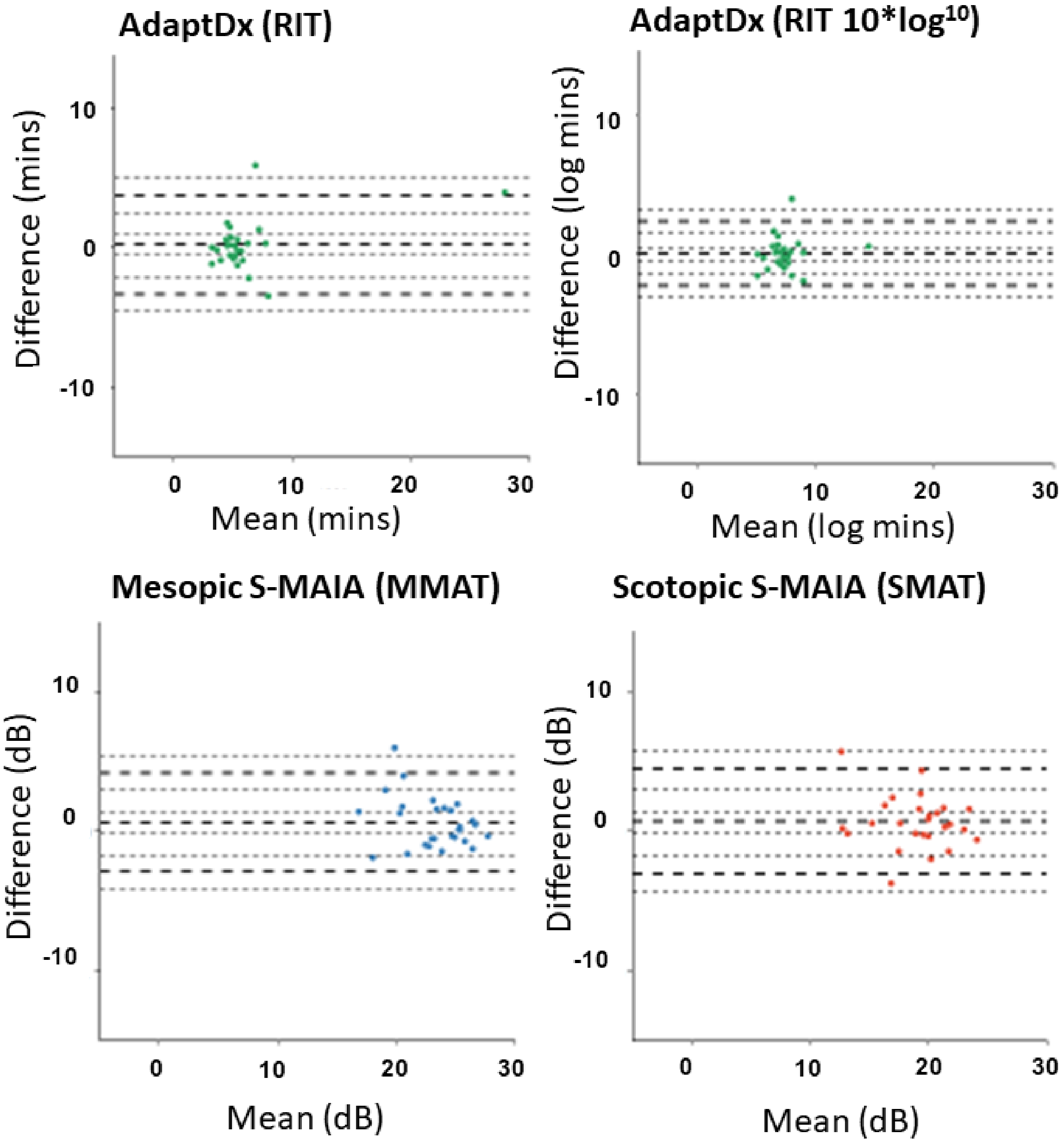

Figure S5. Bland-Altman Plots To Show The Test-Retest Agreement For The Three Devices For Participants With Early Age-Related Macular Degeneration (AMD). Note: Rod intercept time (RIT) has been transformed by $10*\log^{10}$ to mimic the logged output of the S-MAIA for better comparison (top-right). Mean mesopic average threshold (MMAT); mean scotopic average threshold (SMAT); Decibels (dB).

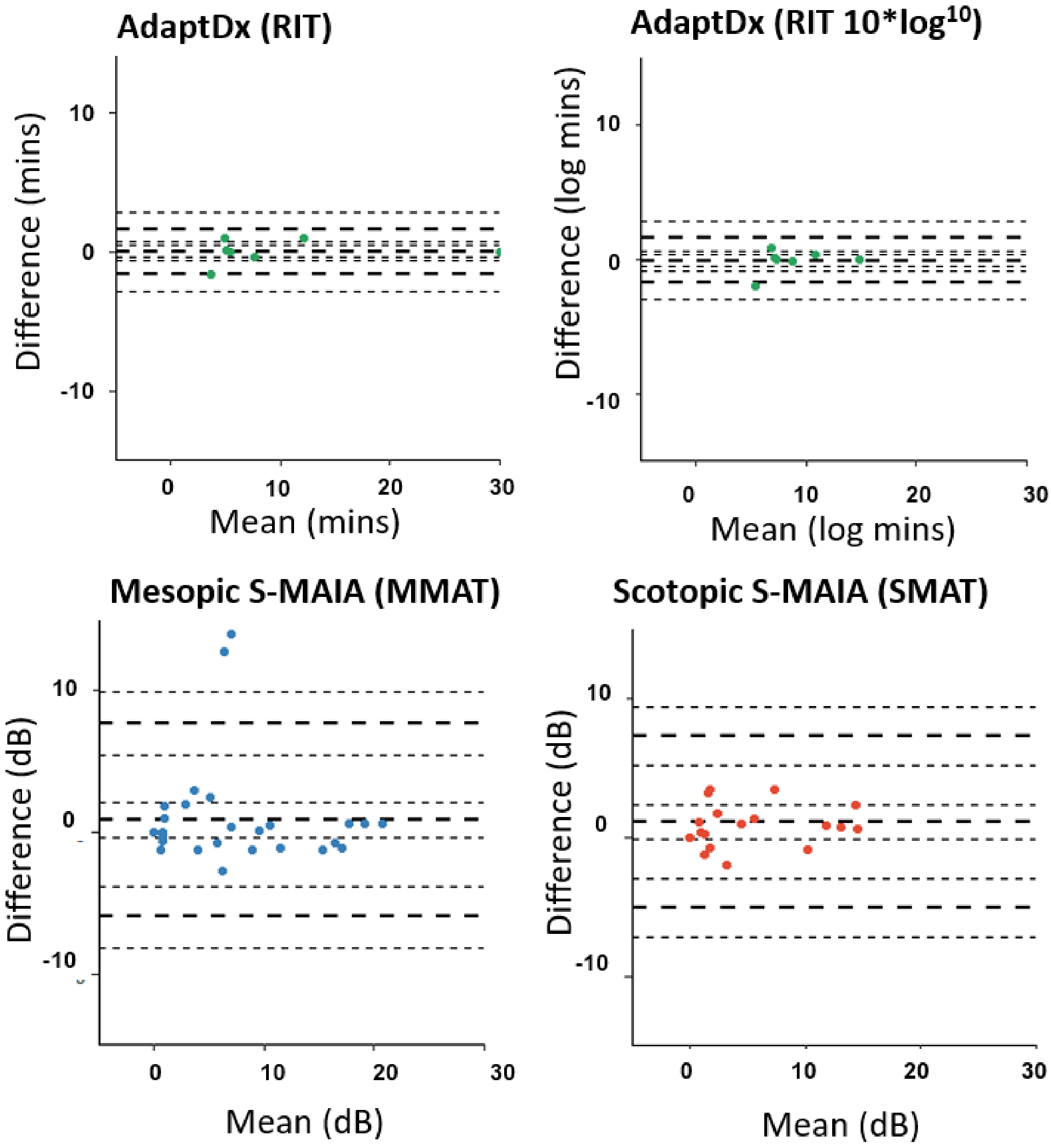

Figure S6. Bland-Altman Plots To Show The Test-Retest Agreement For The Three Devices For Participants With Late Age-Related Macular Degeneration (AMD). Note: Rod intercept time (RIT) has been transformed by $10*log^{10}$ to mimic the logged output of the S-MAIA for better comparison (top-right). Mean mesopic average threshold (MMAT); mean scotopic average threshold (SMAT); Decibels (dB).

Supplemental Tables

Table S1. Intraclass Correlation (ICC) And Bland Altman (Mean Deviation And 95% Limits Of Agreement [LoA]) Metrics For Controls, Early Age-Related Macular Degeneration (AMD), Intermediate AMD (iAMD) and Late AMD Cohorts for AdaptDx And S-MAIA To Demonstrate Cross-Centre Comparison

Test	Centre Location	n at baseline	n removed after screening of procedural errors (%)	n removed after screening of participant issues (%)	n removed after screening of unreliable data (%)	Final n	Bias (95% CI)	Lower LoA (95% CI)	Upper LoA (95% CI)	Interclass Correlation Coefficient (95% CI)	Variability Ratio
**RIT (mins)**	CS001	29	-	1 (6%)	-	28	-0.14 (-0.84, 0.56)	-3.68 (-4.89, -2.47)	3.46 (2.20, 4.72)	0.93 (0.86, 0.97)	0.38
	CS011	11	-	-	-	11	1.81 (-1.79, 5.41)	-8.69 (-15.05, -2.34)	12.32 (5.96, 18.67)	0.52 (-0.05, 0.84)	1.10
	CS015	12	-	-	1 (8%)	11	1.59 (-0.82, 4.00)	-5.44 (-9.70, -1.20)	8.63 (4.37, 12.88)	0.47 (-0.11, 0.82)	1.14
	CS030	12	-	1 (8%)	1 (8%)	10	-1.10 (-2.15, -0.04)	-3.98 (-5.84, -2.12)	1.79 (-0.08, 3.65)	0.94 (0.80, 0.99)	0.28
	Remaining (n14 centres)	103	15 (15%)	1 (1%)	44 (43%)	43	0.95 (-0.42, 2.32)	-7.78 (-10.42, -5.42)	9.68 (7.32, 12.03)	0.51 (0.25, 0.7)	1.13
**MMAT (dB)**	CS001	29	-	-	-	29	0.92 (-0.66, 2.49)	-7.20 (-9.92, -4.48)	9.03 (6.31, 11.76)	0.82 (0.66, 0.91)	0.62
	CS006	10	-	-	-	10	-0.42 (-1.57, 0.73)	-3.58 (-5.63, -1.54)	2.74 (0.70, 4.79)	0.75 (0.30, 0.93)	0.76
	CS011	11	-	-	-	11	-0.17 (-0.66, 0.32)	-1.6 (-2.46, -0.74)	1.25 (0.39, 2.11)	0.92 (0.73, 0.98)	0.42
	CS017	16	-	-	-	16	-0.62 (-1.32, 0.08)	-3.18 (-4.40, -1.96)	1.94 (0.73, 3.16)	0.88 (0.69, 0.96)	0.47
	CS024	11	1 (9%)	-	-	10	0.47 (-0.32, 1.27)	-1.71 (-3.12 - 0.30)	2.65 (1.24, 4.06)	0.87 (0.58, 0.97)	0.51

	CS030	12	1 (8%)	-	-	11	-0.21 (-0.65, 0.23)	-1.49 (-2.26, -0.71)	1.07 (0.30, 1.84)	0.94 (0.81, 0.98)	0.35
	CS077	12	-	-	-	12	-0.46 (-1.13, 0.21)	-2.52 (-3.69, -1.34)	1.60 (0.43, 2.77)	0.69 (0.25, 0.9)	0.81
	Remaining (n11 centres)	66	36 (55%)	-	1 (2%)	29	0.76 (-0.41, 1.92)	-5.24 (-7.25, -3.23)	6.75 (4.74, 8.76)	0.75 (0.54, 0.88)	0.74
**SMAT (dB)**	CS001	29	-	1 (3%)		29	1.34 (0.24, 2.43)	-4.30 (-6.19, -2.41)	6.97 (5.08, 8.86)	0.82 (0.66, 0.91)	0.58
	CS006	10	-	-	-	10	0.13 (-0.68, 0.94)	-2.08 (-3.51, -0.65)	2.34 (0.91, 3.77)	0.65 (0.12, 0.90)	0.97
	CS011	11	1 (9%)	-	-	10	1.07 (-1.22, 3.36)	-5.19 (-9.24, -1.15)	7.33 (3.29, 11.38)	0.33 (-0.31, 0.78)	1.41
	CS017	16	1 (6%)	-	-	15	-0.31 (-1.20, 0.59)	-3.48 (-5.04, -1.91)	2.86 (1.30, 4.43)	0.83 (0.59, 0.94)	0.61
	CS020	20	6 (30%)	-	-	14	0.52 (-0.25, 1.29)	-2.09 (-3.43, -0.74)	3.13 (1.78, 4.47)	0.87 (0.66, 0.96)	0.50
	CS030	12	1 (8%)	-	-	11	0.04 (-0.88, 0.95)	-2.63 (-4.24, -1.02)	2.70 (1.09, 4.31)	0.92 (0.74, 0.98)	0.44
	Remaining (n12 centres)	69	26 (38%)	-	1 (1%)	42	0.02 (-0.76, 0.80)	-4.89 (-6.23, -3.54)	4.92 (3.58, 6.27)	0.84 (0.73, 0.91)	0.59

Table S2. Intraclass Correlation (ICC) And Bland Altman (Mean Deviation And 95% Limits Of Agreement [LoA]) Metrics For Controls Participants for AdaptDx And S-MAIA. Decibels (dB); standard deviation (SD); follow-up (FU); confidence interval (CI).

Test	N	Mean Baseline (±SD)	Mean FU (±SD)	Bias (95% CI)	SD of differences	Lower LoA (95% CI)	Upper LoA (95% CI)	Interclass Correlation Coefficient (95% CI)	Variability Ratio
RIT (mins)	33	4.39 (1.41)	4.14 (0.97)	0.25 (-0.21, 0.71)	0.25	-2.29 (-3.08, -1.5)	2.78 (1.99, 3.58)	0.42 (0.10, 0.66)	1.27
RIT (10*log10)	33	6.20 (1.47)	6.05 (1.05)	0.15 (-0.31, 0.60)	1.29	-2.38 (-3.17, -1.59)	2.67 (1.88, 3.46)	0.50 (0.20, 0.72)	1.17
MMAT (dB)	51	25.28 (2.06)	25.58 (2.11)	-0.3 (-0.72, 0.12)	1.48	-3.2 (-3.91, -2.49)	2.6 (1.89, 3.32)	0.74 (0.60, 0.85)	0.76
S MAT (dB)	49	21.08 (2.46)	20.49 (2.33)	0.59 (-0.0, 1.2)	2.10	-3.53 (-4.57, -2.49)	4.71 (3.68, 5.75)	0.60 (0.38,0.75)	0.98

Table S3. Intraclass Correlation (ICC) And Bland Altman (Mean Deviation And 95% Limits Of Agreement [LoA]) Metrics For Early Age-Related Macular Degeneration (AMD) Participants for AdaptDx And S-MAIA. Decibels (dB); standard deviation (SD); follow-up (FU); confidence interval (CI).

Test	N	Mean Baseline (±SD)	Mean FU (±SD)	Bias (95% CI)	SD of difference	Lower LoA (95% CI)	Upper LoA (95% CI)	Interclass Correlation Coefficient (95% CI)	Variability Ratio
RIT (mins)	26	6.26 (5.08)	6.05 (4.32)	0.20 (-0.52, 0.92)	1.79	-3.30 (-4.56, -2.05)	3.72 (2.46, 4.97)	0.93 (0.85, 0.97)	0.39
RIT (10*log10)	26	7.38 (1.93)	7.33 (1.78)	0.05 (-0.42, 0.52)	1.17	-2.24 (-3.06, -1.42)	2.33 (1.52, 3.15)	0.81 (0.62, 0.91)	0.66
MMAT (dB)	28	23.56 (2.66)	22.94 (3.19)	0.62 (-0.08, 1.32)	1.80	-2.91 (-4.12, -1.7)	4.15 (2.95, 5.36)	0.80 (0.61, 0.9)	0.64
S MAT (dB)	26	19.3 (3.09)	18.62 (3.44)	0.68 (-0.09, 1.45)	1.90	-3.05 (-4.38, -1.72)	4.41 (3.08, 5.74)	0.82 (0.64, 0.91)	0.61

Table S4. Intraclass Correlation (ICC) And Bland Altman (Mean Deviation And 95% Limits Of Agreement [LoA]) Metrics For Late Age-Related Macular Degeneration (AMD) Participants for AdaptDx And S-MAIA. Decibels (dB); standard deviation (SD); follow-up (FU); confidence interval (CI).

Test	N	Mean Baseline (±SD)	Mean FU (±SD)	Bias (95% CI)	SD of difference	Lower LoA (95% CI)	Upper LoA (95% CI)	Interclass Correlation Coefficient (95% CI)	Variability Ratio
RIT (mins)	8	12.37 (11.25)	12.35 (11.16)	0.02 (-0.68, 0.72)	0.83	-1.61 (-0.37, -2.85)	1.65 (0.41, 2.89)	1.00 (0.99, 1.00)	0.07
RIT (10*log10)	8	9.45 (3.76)	9.55 (3.52)	-0.10 (-0.81, 0.61)	0.85	-1.77 (-3.04, -0.50)	1.56 (0.29, 2.83)	0.98 (0.9, 1.00)	0.23
MMAT (dB)	32	6.66 (6.79)	5.81 (6.89)	0.85 (-0.40, 2.10)	3.47	-5.95 (-8.12, -3.79)	7.65 (5.49, 9.82)	0.87 (0.75, 0.93)	0.52
S MAT (dB)	29	4.3 (5.89)	3.21 (4.66)	1.09 (-0.11, 2.28)	3.15	-5.08 (-7.15, -3.01)	7.25 (5.18, 9.32)	0.81 (0.64, 0.91)	0.62

Table S5. Receiver Operating Characteristic Curves (ROC) Area Under The Curve (AUC) Comparing Discrimination Performance Of The AdaptDX Individual Ability To Separate Healthy Controls (No Age-Related Macular Degeneration [AMD]), Early Stage AMD, Intermediate AMD and Late AMD Using Baseline Data. 95% confidence intervals for each curve are provided.

	Controls	Early AMD	Intermediate AMD
Early AMD	73% (59%-86%)	-	-
Intermediate AMD	71% (61%-80%)	55% (43%-66%)	-
Late AMD	82% (61%-100%)	70% (46%-94%)	63% (39%-88%)

Table S6. Receiver Operating Characteristic Curves (ROC) Area Under The Curve (AUC) Comparing Discrimination Performance Of The Mesopic S-MAIA Individual Ability To Separate Healthy Controls (No Age-Related Macular Degeneration [AMD]), Early Stage AMD, Intermediate AMD and Late AMD Using Baseline Data. 95% confidence intervals for each curve are provided.

	Controls	Early AMD	Intermediate AMD
Early AMD	70% (57%-82%)	-	-
Intermediate AMD	68% (60%-77%)	50% (38%-62%)	-
Late AMD	100% (99%-100%)	99% (97%, 100%)	97% (95%-100%)

Table S7. Receiver Operating Characteristic Curves (ROC) Area Under The Curve (AUC) Comparing Discrimination Performance Of The Scotopic S-MAIA Individual Ability To Separate Healthy Controls (No Age-Related Macular Degeneration [AMD]), Early Stage AMD, Intermediate AMD and Late AMD Using Baseline Data. 95% confidence intervals for each curve are provided.

	Controls	Early AMD	Intermediate AMD
Early AMD	66% (53%-79%)	-	-
Intermediate AMD	69% (60%-77%)	53% (40%-65%)	-
Late AMD	99% (96%-100%)	97% (92%-100%)	96% (91%-100%)

Table S8. Receiver Operating Characteristic Curves (ROC) Area Under The Curve (AUC) Comparing Discrimination Performance Of The AdaptDx and Mesopic and Scotopic S-MAIA Individual Ability To Separate Controls (No Age-Related Macular Degeneration [AMD]) From All Participants With AMD and To Separate Healthy Controls From Participants With Iamd Who Completed All Three Tests. 95% confidence intervals for each curve are provided. *31 controls and 81 iAMD participants completed all three tests

	Controls versus all participants with AMD	Controls versus participants with iAMD who completed all three tests*
RIT (mins)	73% (67-79)	70% (60%-80%)
MMAT (dB)	76% (71%-80%)	61% (50%-72%)
SMAT (dB)	72% (67%-77%)	67% (56%-78%)

Supplemental Files

EVICR.net EUROPEAN VISION CLINICAL RESEARCH	**Standard Operating Procedures**	macustar
SOP: VF08	**S-MAIA MICROPERIMETRY**	
Version: 2	Date: 2020/12/10	Replaces Version: 1
Related SOP(s): PM1 – Retinal Imaging – Standard Modalities		

1 Approval:	Name	Date	Signature
Author:	Hannah Dunbar, UCL	2020/12/10	
Reviewed: (GCP compliance review)	Cecília Martinho, AIBILI	2020/12/10	
Authorised:	Frank Holz, UKB	2020/12/10	

Distribution

This SOP is part of the Manual of Study Procedures for the Clinical Study MACUSTAR and its distribution is performed by AIBILI in a controlled manner.

Table of Contents

1. purpose 16
2. policy/scope 16
3. STAFF TRAINING AND RESPONSIBILITIES 16
4. PROCEDURE 18
4.1 Equipment 18
4.1.1 Equipment Maintenance 19
4.2 Subject Information 19
4.3 Technical Procedure 19
4.3.1 Considerations before starting tests 19
4.3.2 S-MAIA Set up for Mesopic testing 20
4.3.3 Preparing the subject 24
4.3.4 Description of Mesopic Procedure 25
4.3.5 Reliability checks 30
4.3.6 S-MAIA set up for Scotopic Procedure 31
4.3.7 Description of Scotopic procedure 34
4.3.8 Reliability checks 35
4.3.9 Labelling data and data exportation 36

4.3.10 Data backup 38
4.3.11 End of test 38
4.4 Storage 38
5. list of attachments 39

1. PURPOSE

This document describes the procedures to perform S-MAIA microperimetry for the Clinical Study *"Development of novel clinical endpoints for interventional clinical trials with a regulatory and patient access intention in patients with intermediate age-related macular degeneration (AMD) – MACUSTAR"*(Protocol nº ECR-AMD-2017-13) to ensure that a uniform procedure is followed by all clinical sites (CS) participating in the study, in order to obtain comparable and reliable data, as according to International Conference on Harmonization Good Clinical Practice (ICH-GCP). This procedure will be performed to the study subjects according to the Clinical Study Protocol.

2. POLICY/SCOPE

This SOP will be used when performing all microperimetry assessments (Mesopic, Scotopic Red) using the S-MAIA Microperimeter to ensure the procedures are performed consistently at all visits and across all clinical sites. Adherence to this SOP will ensure accurate measurement and that all data are available for analysis.

Changes in the Clinical Study Protocol may lead to new versions of this SOP.

If the need for modification or withdrawal of the SOP or Attachment is identified, ensure that this information is reported in writing to the author of this SOP, Hannah Dunbar (h.dunbar@ucl.ac.uk) who will decide on the need of revision.

The Coordination Centre, AIBILI, ensures the SOP is distributed to all that may be concerned.

SOPs must be written in English.

3. STAFF TRAINING AND RESPONSIBILITIES

The Principal Investigator is responsible for ensuring that the appropriate personnel for performing S-MAIA Microperimetry is identified and trained. All technicians should read this procedure before starting the participation in the Study. Clinical Sites are recommended to have a minimum of 2 technicians in the investigational team certified for this procedure.

Technicians will be certified as follows:

1) Basic training in using the device will be provided by the manufacturer on installation.
2) Each technician will read this SOP and the Microperimetry: Pearls and Pitfalls – att. VF08-1. Each technician will perform one new and one follow up S-MAIA Scotopic Red exam on one person without retinal pathology and one person with AMD. Mesopic testing is not required for the purposes of certification. If data are collected which the technician feels are unreliable, they should repeat the test. If a repeat test is required, and as long as the subject is still dark adapted, the repeat test can be performed immediately. Technicians should complete one Dark Adaptation Set Up Form – att. V08-2 for each subject tested. Technicians should also complete the Microperimetry Certification Examination via the electronic Certification Portal described below in 8).
3) Certification S-MAIA data should be exported and transferred to GRADE as described in section 4.3.9 via the GRADE Reading Centre Portal. Where a test was repeated, both the original and the repeat test should be submitted.
4) Each technician will also complete the Application for Certification of Microperimetry and AdaptDx– att. VF08-3 for submission to GRADE. Forms can be filled out electronically or manually with black pen in capital letters to ensure legibility. General data related to study site and contact details of technicians are to be completed in Sections 1 and 2 respectively.
5) Tick 'Centervue S-MAIA' in Section 3 and record the serial number and software version in use. Technicians must provide comments describing their experience of using the S-MAIA and any problems encountered. This report will state whether they consider the test results obtained from each subject to be of high quality and if not, what they consider to be the issues with the data.
6) The Application for Certification of Microperimetry and AdaptDx – att. VF08-3, and Dark Adaptation Set Up Form – att. VF08-2 must be named, dated and signed and sent to GRADE either by fax on +49 228 287 9014813 or scanned via email to macustar@grade-rc.de.
7) Prior to undertaking any certification procedures, technicians should complete the Application for Certification of Photographers / Technicians for Microperimetry and

AdaptDx – att. VF08-5 recording the name and contact details for all technicians requesting to be certified. Tick boxes should be checked indicating the imaging modality(ies) each technician requests certification for. The Application for Certification of Photographers / Technicians for Microperimetry and AdaptDx – att. VF08-5 should be submitted to GRADE either by fax on +49 228 287 9014813 or scanned via email to macustar@grade-rc.de.

8) On receipt of the Application for Certification of Photographers / Technicians for Microperimetry and AdaptDx – att. VF08-5, GRADE will issue a username and 2 passwords to each technician by email; one password for the Certification Portal and one password for the GRADE Reading Centre Portal. Please note, the two passwords will be different, but after initial sign into each portal, technicians will be prompted to change their assigned password and may synchronise the 2 passwords if they wish.
9) Each technician will complete the Microperimetry Certification Examination, a short multiple choice question exam including questions on Mesopic and Scotopic Red microperimetry testing via the Certification Portal (https://test.certification.macustar.eu). This examination must be passed with 100%.
10) Notice of certification will be provided by GRADE by e-mail.

4. PROCEDURE

4.1 Equipment

This procedure requires the use of the following equipment:

S-MAIA Microperimeter (Centervue) running software version 2.5.1
Adjustable chair with back rest
1 watt, 660nm red LED torch (dim red torch)
787 Marius Red plastic filter sheet
Red perspex screen
USB mouse
Encrypted USB key
Tropicamide minims 1% eye drops
Cotton pads

Surgical tape

Alcohol hand rub

Alcohol wipes

4.1.1 Equipment Maintenance

The supplier recommendations for the equipment should be followed for maintenance. Only qualified and trained personnel should do service and repair. All interventions in the equipment should be recorded, kept and made available during monitoring visits or audits if requested.

4.2 Subject Information

Oral information regarding the examination that is being performed is given to the subject.

4.3 Technical Procedure

4.3.1 Considerations before starting tests

a) Dark Adaptation

The room in which this procedure takes place should be completely dark. This includes blocking the output from any LEDs in the test room (e.g. from computers), unless the light output is dim and red. Light should be blocked from entering under closed doors, arrangements should be made to extinguish or block the output of safety lights. '787 Marius Red' plastic sheet filter is recommended to cover any essential lights that cannot be extinguished (available at http://www.leefilters.com/lighting/colour-details.html#787&filter=cf). Only the dim red torch identified in section 4.1 should be used during dark adapted procedures.

The subject should be dark adapted for 5 mins after instillation of 1% tropicamide (see point b) prior to beginning Mesopic microperimetry and by a further 30 mins prior to beginning Scotopic Red microperimetry.

The subject's eye must remain open (blinking permitted) or gently closed during this time. They must not squeeze their eyes shut. The door should be locked from the inside to avoid it being inadvertently opened during dark adaptation or testing. Alternatively a large visible sign should be placed on the outside of the door telling people not to enter. Remind subjects they must not look at mobile

phone, tablet, watch or computer screens while dark adapting. Technicians must also not use mobile phones, tablets or computers while the subject is dark adapting.

b) Pupil dilation

Prior to Mesopic testing, 1 drop of 1% tropicamide should be instilled into both eyes (in preparation for imaging both eyes after dark adapted testing). Subjects should begin mesopic testing 5 minutes after drop instilled. Subjects should be informed that pupil dilation will wear off in 4-6 hours and warned not to drive during this time. Inform subjects to seek medical attention if they experience any discomfort during this time.

c) Testing procedure

Only the study eye will be tested. Mesopic microperimetry will be followed by a further 30 minutes of dark adaption, after which Scotopic Red microperimetry should be performed on the same eye.

d) Practise session

At study visit 2 (baseline), all subjects will perform a 15 point practise session using 'MACUSTAR PRACTISE' grid. The required XML file for this grid can be downloaded from the GRADE portal. A practise session is not required at future study visits.

4.3.2 S-MAIA Set up for Mesopic testing

a) With the room lights on, remove the protective cover and lens cap. Switch on the S-MAIA and wait until the software loads and the Start up screen appears. This should take approximately 1 minute.

b) Cover the illuminated S-MAIA control panel with its red plastic filter. This should remain in place throughout mesopic and scotopic testing.

c) S-MAIA control panel offers touch screen control and can also be operated by USB mouse inserted into an available S-MAIA USB port. If the touch screen fails to accurately recognise a finger press position it may need to be recalibrated. Click on the wheel shaped symbol in the top right corner of the Start up screen and

select ‘Settings’, then ‘System’. Press ‘Calib Touchscreen’. Touch the centre of the symbols that appear in the four corners of the screen to recalibrate the touch screen.

d) The interaction between the touch screen control panel and the red filter can reduce the responsiveness of the touch screen. In this case, use the USB mouse to control the S-MAIA.

e) Wipe the chin rest, headrest and response button with an alcohol wipe. The subject will appreciate witnessing your attempts to provide them with a clean and sterile testing environment.

f) Instill 1 drop of 1% tropicamide to each eye. Switch off the room lights. Note the time when the lights were extinguished and record it on the Dark Adaptation Set Up Form – att. VF08-2.

g) The room should remain dark throughout all remaining set-up steps and testing to avoid light adapting the subject. A dim red torch may be used for moving around the room. Only the dim red torch identified in section 4.1 should be used. Subjects and examiners must not use mobile phones, or other illuminated devices whilst the subject adapts.

h) Advise the subject of the purpose of adapting and remind them to keep their eyes open (blinking permitted) or gently closed and not to squeeze their eyes shut.

i) Whilst the subject dark adapts, enter subject details:

During a baseline visit: On the start up screen, press the ‘NEW PATIENT’ button on the top right of the screen. The subject’s identification code should be inputted into all participant name fields. Date of birth should be entered as 1900-01-01 for all subjects. Subject’s true birth dates should not be used. The subject’s Patient Record Screen will be displayed. Press ‘NEW EXPERT EXAM’.

During a follow up visit: On the start up screen, enter the subject’s identification code in the search field on the top left of the screen. Click on the correct subject. The subject’s Patient Record Screen will be displayed. Select the most recent MACUSTAR mesopic assessment and press ‘FOLLOW-UP EXAM’. Be careful not to select a scotopic exam.

j) The Test Screen will now be displayed. The S-MAIA control panel will default to a dark background and the 'Colors: Dark' button on the bottom right corner of the screen will be highlighted. No other background is permitted. To the left of this button, the 'Tracking Alarm' should be highlighted blue.

k) In the box labelled 'Selected Projection Grid' on the bottom right of the screen, ensure 'Condition: Mesopic' is displayed. Ensure 'Strategy' is set to 4-2. These steps ensure the correct testing parameters are used and are essential for the accuracy of the assessment. An image of correctly adjusted Test Screen is shown in Figure 1. Please note 'MACUSTAR' grid layout is not represented accurately in the image below.

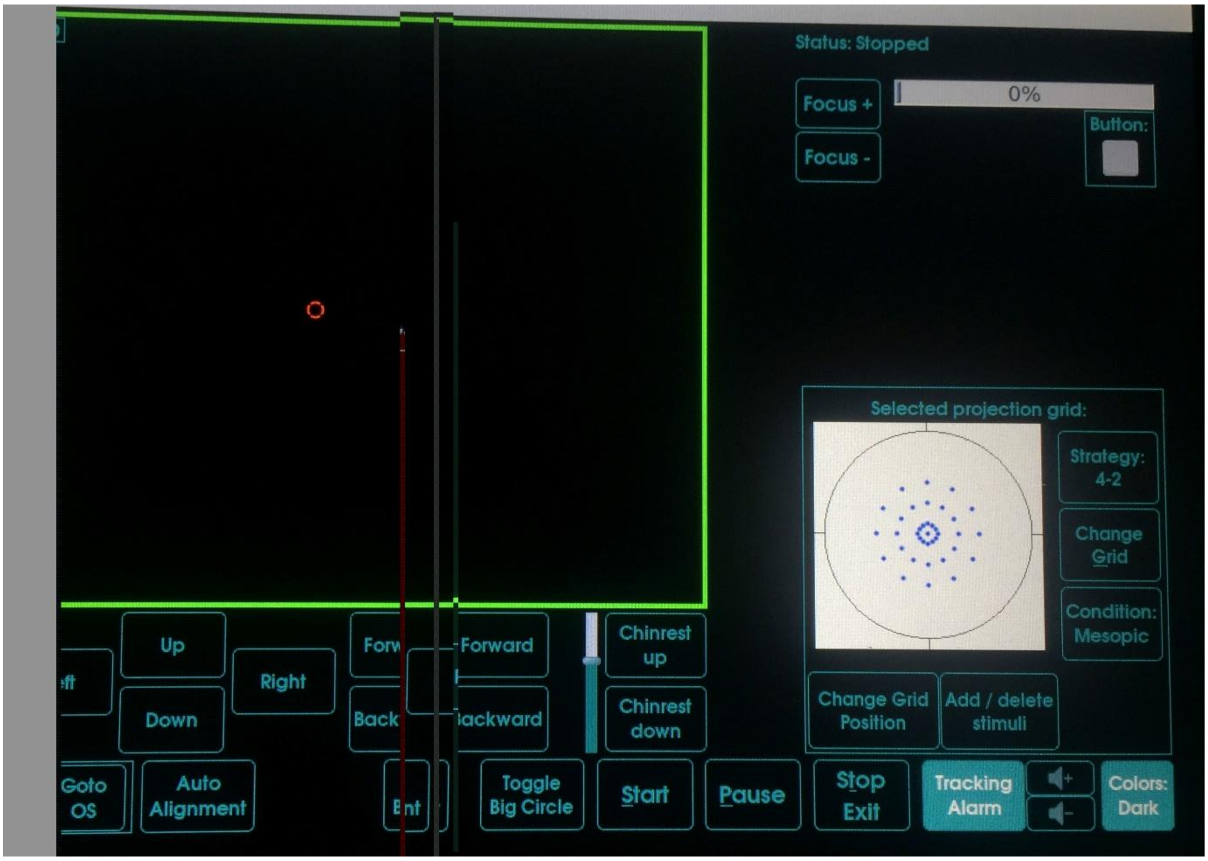

Figure 1: Test screen for Mesopic testing

l) Press ‘Change grid’ and select ‘MACUSTAR’. The parameters of the ‘MACUSTAR’ grid in mesopic conditions are as described in table 1. The required XML file for this grid can be downloaded from the GRADE portal.

MACUSTAR GRID PARAMETERS	
Number of Stimuli	33
Stimulus size	Goldmann III
Stimulus duration	200 m/s
Threshold strategy	4-2 staircase
Background luminance	1.27 cd/m^2
Initial target brightness	2.6 ± 0.5 asb
Stimulus configuration	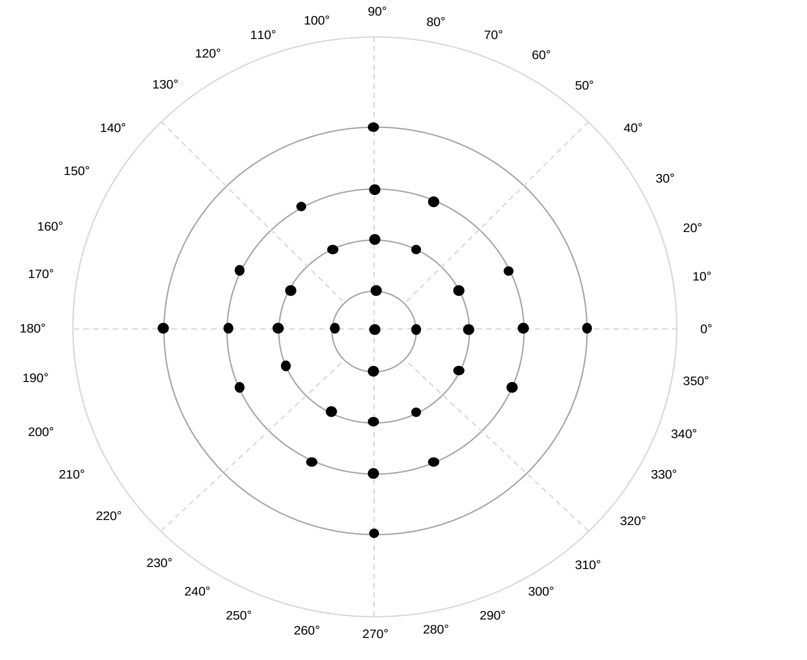

Table 1: MACUSTAR Mesopic microperimetry grid parameters

m) If a practice session is required (see 4.3.1.d), select 'MACUSTAR PRACTISE' grid and follow the same procedure below. When the practice session is completed, repeat the procedure below, being sure to select the 'MACUSTAR' grid and start a 'NEW EXPERT EXAM'.

4.3.3 Preparing the subject

a) Seat the subject facing the S-MAIA chin rest. Instruct the subject to bring their chair as close to the S-MAIA as possible, place their chin on the chin rest and press their forehead against the headrest. If the chin rest is too high move the table down. If the chin rest is too low, move the table up. Next adjust the chin rest position using the 'Chinrest up' and 'Chinrest down' button on the touch screen to

align the corner of the subject’s eye with the mark on headrest. Readjust the table height again if necessary. The subject should feel as though they are slightly falling into the instrument. Take sufficient time to position to the patient correctly as the quality of the examination is reliant upon the subject being able to keep a steady position for the duration of the test. Once the table, chin and headrest position have been determined, the subject may sit back from the S-MAIA whilst you explain the procedure.

b) Rotate the instrument mounted adjustable occluder to cover the non-study eye. Apply a cotton pad over the eye not being tested and hold in place with surgical tape.
c) Give the response button to the subject.
d) Give the subject oral information (see 4.3.4).
e) Remove any spectacles from the subject.

4.3.4 Description of Mesopic Procedure

a) Explain the procedure to the subject. “This test measures how well you detect light in your central vision when your eyes have been adapted to dim light. In a moment I will ask you to place your chin on the rest in front of you and your forehead against the headrest. When you look into the instrument you will see a small round red target on the black background. For the duration of this test I would like you to look directly at the round target. When the examination starts a series of small spots of light will flash on the black background around the circle, one at a time. The small spots of light will look white. Each time you see a light you should press the button in your hand. You must not move your eye to find these lights, but rather you will notice them in your side vision. Some lights will appear close to the target. Some will appear further from the target. The shape of the lights may differ. Lights in the centre can appear round and well-demarcated, while lights further from the centre can appear star-shaped. Regardless of what shape the light is, if you see a light you should press the button. The intensity (brightness) of the lights will vary. Some may appear brighter than others, but you should press the button for each light you see. Even if a light appears very dim you should press the button. We are trying to find the intensity of the dimmest light you can see, therefore the light will sometimes be too dim to be seen

and you will not see a light. This is a normal part of the test and you should not let it worry you. It is very important that you keep your eye on the circle target throughout the test. The instrument automatically measures how well you look at the circle throughout the test. If you move your eye too much, the results of the test will be inaccurate. You should blink when you need to, but try to keep your eye open wide between blinks. It can be useful to blink after you respond to a light. You can ask me for a break at anytime throughout the test. Do you understand?”

b) The subject should confirm they have understood the above before proceeding. If they have any questions or seek further clarification, respond accordingly. If they do not understand the procedure, repeat step a). Only move onto step c) when the subject confirms they have understood.

c) Instruct the subject to place their chin on the chin rest and forehead against the headrest.

d) Instruct the patient “I will now position the instrument so that you can see the red circle target. The instrument will move right and left, towards you and away from you whilst it finds the best position. Please try to keep your head and eyes still and look forward during this procedure. Do not follow the instrument with your eye”.

e) If testing the right eye, press ‘OD’ on the bottom left corner of the touch screen (Figure 1). If testing the left eye, press ‘OS’ on the bottom left corner of the touch screen. The instrument will automatically move to the corresponding eye. When the instrument has stopped moving, press ‘Auto Alignment’ to the right of the ‘OS’ button. The instrument will move forward and back as it tries to find the optimal infrared reflectance view of the fundus, displayed in the box on the top left of the set up screen.

f) Adjustments to the infrared reflectance image can be made using the ‘Up’, ‘Down’, ‘Left’, ‘Right’, ‘Forward’ and ‘Backwards’ buttons below the infrared reflectance image on the touch screen (Figure 1). Final adjustments can be made using the ‘Focus +’ and ‘Focus –’ buttons to the top right of the infrared reflectance image if required. It is particularly important that the vessels in the centre of the image are in sharp focus. The infrared reflectance image should fill the screen with the macula in the centre of the image and optic disc visible. Three vessel bifurcations in proximity to the fovea must be visible in the infrared reflectance image. If the instrument is too far away,

the fundus image will not fill the screen. If the instrument is too close, distortion of the fundus image will appear. Follow the on screen adjustment prompts during setup. If the auto alignment function is incorrectly aligning the instrument, press ‘Manual Focus’, and use the ‘+’ and ‘-’ focus keys to manually adjust the focus. Examples of acceptable and unacceptable images are shown in the Microperimetry: Pearls and Pitfalls – att. VF08-1.

g) Remind the subject they must not move their eye or head during the alignment process. As soon as they report seeing the red target they should look toward it. If the subject cannot see the red fixation target despite it being centred on the macula, increase the fixation target power (brightness) in a step-wise manner by pressing ‘+’ symbol below ‘Power’ until the subjects reports the target is seen. Confirm the subject is looking at the target by viewing the infrared reflectance image. The minimum power consistent with the subject being able to see the target should be used and recorded on the Dark Adaptation Set Up Form – att. VF08-2.

h) If the subject cannot see the small red circle fixation target despite increasing the power, change the fixation target to four crosses by pressing the button below ‘Fix. Target’ (Figure 1). Four crosses will now be seen in the button, confirming the fixation target has been changed. Reduce the power to its lowest setting and ask the subject if they can see the four crosses. If they can, direct them to look at the centre of crosses. If the subject cannot see the four crosses, increase the power (brightness) of the crosses as described in g). The minimum power consistent with the subject being able to see the four crosses should be used and recorded Dark Adaptation Set Up Form – att. VF08-2.

i) Only if a subject is in the Late AMD group and cannot see either the small red target or the four red crosses despite increasing the power, press ‘Toggle Big Circle’(Figure 1). This displays a large ring around the target. The subject should direct their gaze to the centre of this large circle. If this is not possible, guide the subject’s gaze verbally based on the fundus image on the screen.

j) Regardless of the fixation target used, continue to verbally guide the subject throughout testing.

k) Check the response button is functional before commencing the test. Give the response button to the subject and ask them to press it. If the response button is functional, the ‘Button’ indicator on the test screen will turn orange as the response button is pressed. If the response button is not functional, patient responses will not be detected throughout testing and incorrect threshold values will be recorded. Furthermore, during the examination ensure that each ‘click’ sound by the response button is accompanied by the ‘button’ indicator turning. If the response button is not functional, testing cannot take place and a protocol violation should be recorded. The Coordinating Centre must be informed within 24 hours.

l) Check that the subject has dark adapted for the necessary amount of time. If so, instruct the subject that the test is about to start. Record the time the test was started on the Dark Adaptation Set Up Form – att. VF08-2. Press ‘Start’ at the bottom centre of the screen. Once ‘Start’ is pressed, an additional auto focus will occur as the S-MAIA captures one retinal image for reference. This takes approximately 10 seconds. You will be prompted to accept or reject the resultant image. Accept the image if it is clear and in focus, fills the viewing window, the macula is in the centre of the image, the optic disc is visible and 3 vessel bifurcations in proximity to the fovea can be clearly seen; otherwise reject the image. If the image is rejected, press ‘Run Auto-focus’ to repeat this step. If after a second attempt an acceptable image is not achieved, press ‘Manual focus’ and use the ‘+’ and ‘-’ focus keys to manually adjust the focus.

m) You will be prompted to select the centre of the optic disc. Press ‘OK’ once this has been done. Inaccurate identification of the optic disc centre will result in erroneous false positive fixation errors, which may result in the test results being falsely considered inaccurate and the test needing to be repeated.

n) During the first 10 seconds of the test, the S-MAIA will record the subject’s fixation (approximately 250 points). The centre of fixation defined during these 10 seconds is used to estimate the preferred retinal locus (PRL). In new expert exam mode, the S-MAIA automatically centres the grid on the PRL. This approach is appropriate for all subjects in the normal, early AMD and intermediate AMD groups, but not for subjects in the late AMD group. During all new expert exams in subjects in the late AMD group, the ‘Change grid’ button should be pressed and the ‘MACUSTAR’ grid manually placed

at the foveal centred based on SD-OCT imaging. Refer to New Expert Exam MACUSTAR grid placement in subjects with late AMD – att. VF08-6 for detailed instruction on how this is achieved. Please note, in this situation a change to the order of testing will be required to allow for SD-OCT imaging to occur before Microperimetry and for the necessary print out to be available. In follow-up exam mode, for subjects in all disease groups, the S-MAIA will automatically centre the grid to ensure retinal sensitivity is measured in the same retinal locations across study visits.

o) Once the grid has been positioned, microperimetry will begin. The S-MAIA will track the position of the eye throughout testing by monitoring the infrared reflectance image. If the perimeter of the infrared reflectance image is green, the eye position is being tracked. If the perimeter of the infrared reflectance image is red, eye position is not being tracked and the tracking alarm will sound indicating the subject's eye and or head has moved. No testing occurs when the S-MAIA is unable to track. Remind the subject to place their chin on the rest, their forehead firmly against the headrest and to look directly at the target. If tracking does not resume automatically, use the 'Up', 'Down', 'Left', 'Right' buttons below the infrared reflectance image on the touch screen to realign the image. Forward and back buttons must not be used during testing. If tracking cannot be resumed, the test should be abandoned and restarted.

p) Encourage the subject throughout testing, reminding them to maintain their head and eye position, to blink after pressing the response button but keep their eyes wide between blinks and to let you know if they need a break.

q) If the patient requests a break or if you suspect the subject needs a break, press the 'Pause' button to the right of the 'Start' button at the bottom of the screen (Figure 1). When the test has paused, instruct the subject to sit back from the instrument. When restarting testing, ask the subject to place their chin on the rest and their forehead against the headrest as before. Make sure the border of the infrared reflectance image turns green before resuming testing. Ask the subject if they are ready to resume. Only start when the subject verbally confirms they are ready. Advise the subject you will count them down from 3 and the test will begin as you say the word start. Clearly count the patient down as follows by saying: "Three, Two, One, Start". You must press the 'Start' button as you say the word start (Figure 1).

r) The test will continue in an automated fashion until completed. The examiner should monitor the following throughout the test:
    - ‘Remaining stimuli’ left, giving subject feedback as encouragement
    - Ensure button indicator turns orange when response button pressed. Refer to k) if it does not.
    - Monitor the infrared reflectance image to ensure tracking in process and refer to step o) if tracking is lost.

s) On completion of the test, a message will appear on the screen reporting ‘Exam finished’.

t) The Expert Test Results page will be displayed as in Figure 2 below.

u) Do not turn on lights when test procedure is finished.

4.3.5 Reliability checks

a) Note the Fixation Losses on the top right of the Expert Test Results page (Figure 2). If Fixation Losses are 30% or greater the test may be inaccurate. If Fixation Losses exceed 30% the test does **not** need to be repeated and should still be submitted to GRADE.

b) If 95% BCEA (Bivariate Contour Ellipse Area) value is greater than 50 deg^2 (Figure 2), the test may be inaccurate. If 95% BCEA exceeds 50 deg^2 the test does **not** need to be repeated and should still be submitted to GRADE.

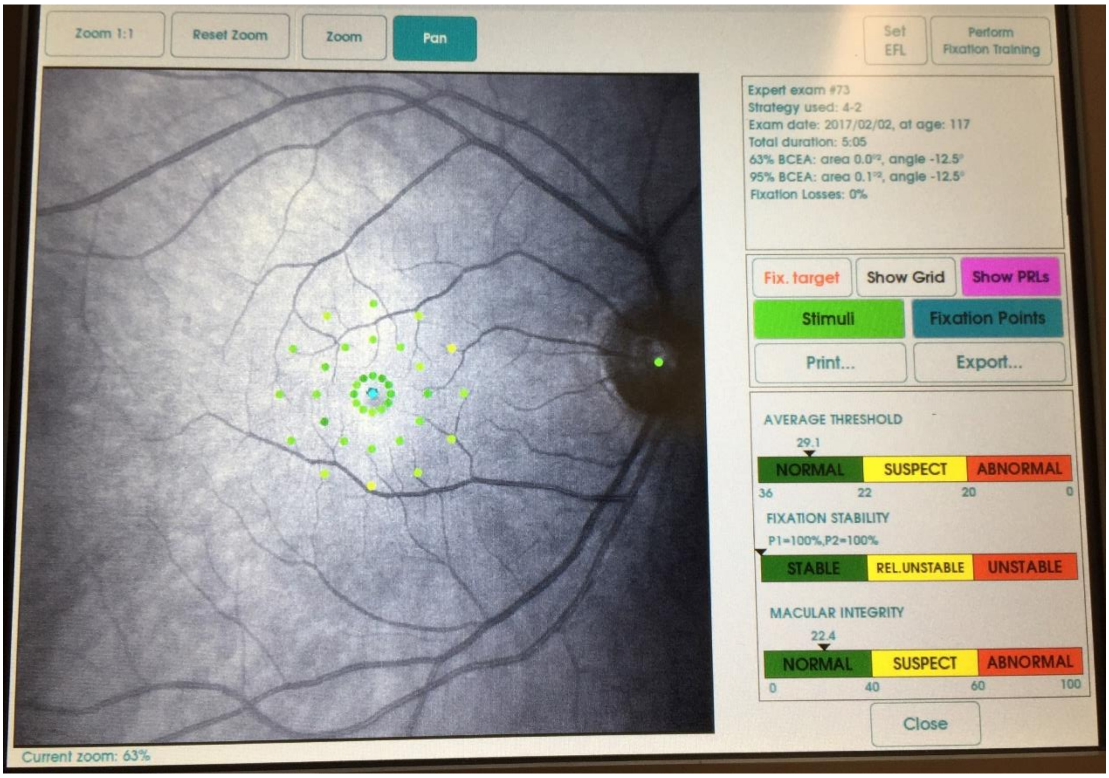

Figure 2: Expert Test Results screen

4.3.6 S-MAIA set up for Scotopic Procedure

a) Prior to Scotopic Red testing, the subject should dark adapt for a further 30 mins in the same conditions as described in 4.3.1 a). Advise the subject of the purpose of adapting and that the process takes 30 minutes. Remind the subject to keep their eyes open (blinking permitted) or gently closed and not to squeeze their eye shut.

b) The room should remain dark throughout all remaining set-up steps and testing to avoid light adapting the subject. A dim red torch may be used for moving around the room. Only the dim red torch approved by the Sponsor should be used. Subjects and examiners must not use mobile phones, or other illuminated devices whilst the subject adapts.

c) Note the time when dark adaption started (after mesopic testing) and record it on the Dark Adaptation Set Up Form – att. VF08-2. and after 30 minutes continue with S-MAIA set up.

d) The PRO Questionnaires (e.g. VILL and EQ-5D-5L)can be administered while the subject dark adapts using the dim red torch for illumination (see MACUSTAR Manual of Procedures).

e) After 30 minutes of dark adaptation, retrieve subject data by entering the MACUSTAR subject identification code in the search bar of the start up screen:

   During a baseline visit: Press ‘NEW EXPERT EXAM’.

   During a follow up visit: Select the most recent MACUSTAR scotopic assessment and press ‘FOLLOW-UP EXAM’. Be careful not to select a mesopic exam.

   During a certification examination: Technicians should perform both a new and follow up Scotopic Red exam on 1 normal eye without retinal pathology (NOR) and 1 person with AMD. In the start up screen, enter ‘NOR’ or ‘AMD’ into all participant name fields during the certification process. Date of birth should be entered as 1900-01-01 for all subjects. Subject’s true birth dates should not be used. During a new exam, select ‘NEW EXPERT EXAM’. During a follow up exam, select the ‘NEW’ exam performed on the Patient Record Screen and press ‘FOLLOW-UP EXAM’.

f) The Test Screen will now be displayed. The S-MAIA control panel will default to ‘a dark background and the ‘Colors: Dark’ button on the bottom right corner of the screen will be highlighted. No other background is permitted. To the left of this button, the ‘Tracking Alarm’ should be highlighted blue (Figure 3).

g) In the box labelled ‘Selected Projection Grid’ on the bottom right of the screen, ensure ‘Condition: Scotopic’ is displayed (Figure 3). Press the ‘Stimulus color’ button until ‘red’ is displayed. Beneath this, press ‘Fix. Target’ until a small circle is displayed. These steps ensure the correct testing parameters are used and are essential for the accuracy of the assessment. An image of correctly adjusted Test Screen is shown in Figure 3.

h) Please note ‘MACUSTAR’ grid layout is not represented accurately in the image below.

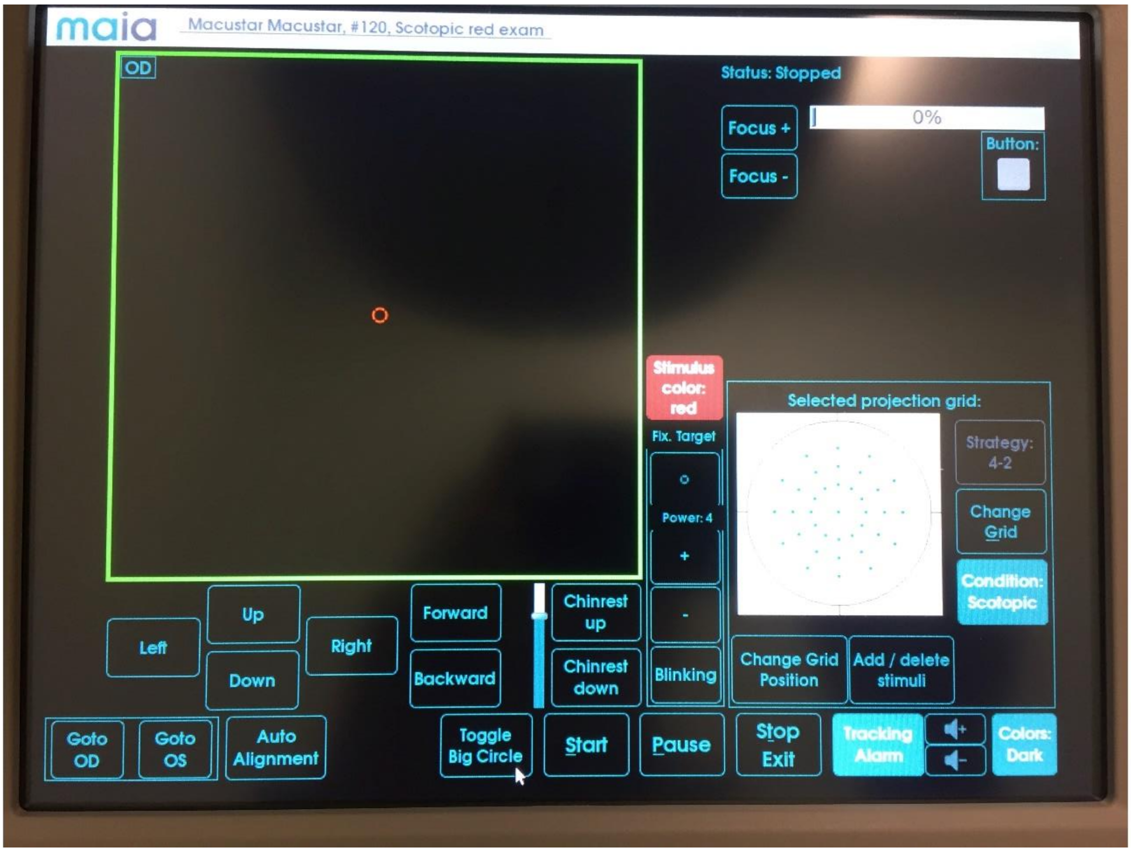

Figure 3: Test screen for Scotopic testing

i) Press ‘Change grid’ and select ‘MACUSTAR’. The parameters of the ‘MACUSTAR’ grid in scotopic conditions are as described in table 2. The required XML file for this grid can be downloaded from the GRADE portal.

MACUSTAR SCOTOPIC GRID PARAMETERS	
Number of Stimuli	33
Stimulus size	Goldmann III
Stimulus duration	200 m/s
Threshold strategy	4-2 staircase
Background luminance	<0.0001 cd/m^2
Initial target brightness	0.01 asb
Stimulus configuration	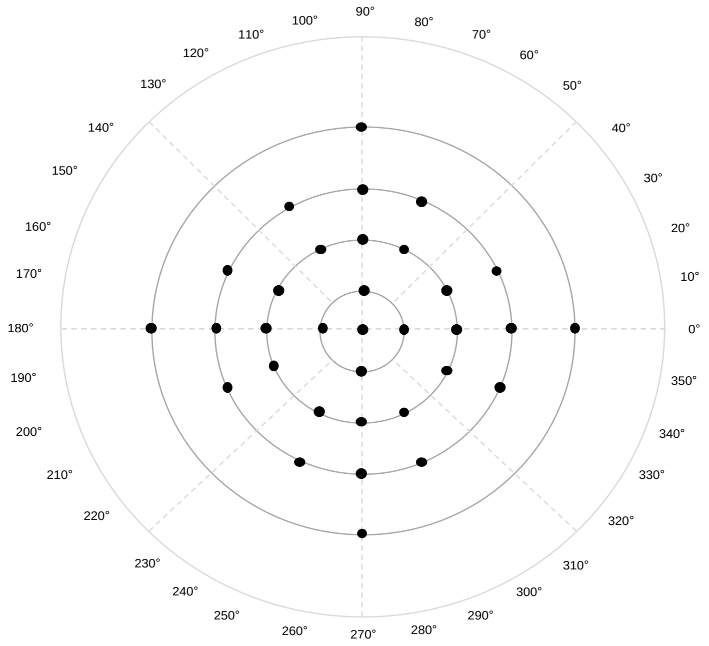

Table 2: MACUSTAR Scotopic microperimetry grid parameters

j) Prepare the subject in the same manner as described in 4.3.3.

4.3.7 Description of Scotopic procedure

a) Explain the procedure to the subject. “This test measures how well you detect light in your central vision when your eyes have been adapted to the dark. In a moment I will ask you to place your chin on the rest in front of you and your forehead against the headrest. When you look into the instrument you will see a small round red target on

the black background. For the duration of this test I would like you to look directly at the round target. When the examination starts a series of small spots of light will flash on the black background around the circle one at a time. The small spots of light will look white or red. Each time you see a light you should press the button in your hand. You must not move your eye to find these lights, but rather you will notice them in your side vision. Some lights will appear close to the target. Some will appear further from the target. The shape of the lights may differ. Lights in the centre can appear round and well-demarcated, while lights further from the centre can appear star-shaped. Regardless of what shape the light is, if you see a light you should press the button. The intensity (brightness) of the lights will vary. Some may appear brighter than others, but you should press the button for each light you see. Even if a light appears very dim you should press the button. We are trying to find the intensity of the dimmest light you can see, therefore the light will sometimes be too dim to be seen and you will not see a light. This is a normal part of the test and you should not let it worry you. It is very important that you keep your eye on the circle target throughout the test. The instrument automatically measures how well you look at the circle throughout the test. If you move your eye too much, the results of the test will be inaccurate. You should blink when you need to, but try to keep your eye open wide between blinks. It can be useful to blink after you respond to a light. You can ask me for a break at anytime throughout the test. Do you understand?"

b) The subject should confirm they have understood the above before proceeding. If they have any questions or seek further clarification, respond accordingly. If they do not understand the procedure, repeat step a). Only move onto step c) when the subject confirms they have understood.

c) Continue the test in the same manner described in 4.3.4 c) to u).

4.3.8 Reliability checks

a) Note the Fixation Losses on the top right of the Expert Test Results page (Figure 2). If Fixation Losses are 30% or greater the test may be inaccurate. If Fixation Losses exceed 30% the test does **not** need to be repeated and should still be submitted to GRADE.

b) If 95% BCEA value is greater than 50 deg^2 (Figure 2), the test may be inaccurate. If 95% BCEA exceeds 50 deg^2 the test does **not** need to be repeated and should still be submitted to GRADE.

4.3.9 Labelling data and data exportation

a) Expert Test results for mesopic exam and scotopic red exam (PDFs) should be transferred to the encrypted USB key and printed.

b) To print, insert an encrypted USB key into the USB drive to the right of the machine. On the Expert Test Results page press 'Print…' and select USB key as the destination (Figure 2). This creates a file named as follows:
MAIA_exam_PID_EXID.pdf where PID is the Patient specific ID allocated by the instrument (not MACUSTAR subject identification code) and shown to the left of the subject's entry on the 'Patient List' screen and EXID is the device specific Exam ID which can be found in to top right of the result page followed by a #. Repeat this step for Mesopic and Scotopic Red exams.

c) The PDFs should be printed and filed in the subject's clinical notes.

d) Make a scanned copy of the participant's Dark Adaptation Set Up Form – att. VF08-2. (PDF).

e) To export S-MAIA raw data, select and open the respective patient in the 'Patient List'. Insert the encrypted USB key into the USB drive to the right of the machine. A USB-symbol will appear in the upper right corner of the screen. By pressing on the USB symbol, you will export the patient data as TGZ formatted file to the USB key.

f) Move all PDFs (S-MAIA results and Dark Adapted Set Up form) and TGZ files into a data folder in preparation for submission to GRADE.

g) Prior to submission to GRADE, all files (PDF and TGZ) and folders must be renamed in accordance with Section 8 of the Procedure Manual 1 (PM1) – Retinal Imaging - Standard Modalities. GRADE may reject submissions with incorrectly labelled files or folders. Rejected data can be resubmitted after correction. File labelling expressions are described in Section 8.1 of the PM1 where the following expressions should be used for S-MAIA:

Modality – Use the following abbreviations for indicating modalities:

S_MAIA (= Centervue MAIA Microperimeter)

S_MAIA_M (= Mesopic examination on S-MAIA instrument)

S_MAIA_R (= Scotopic Red examination on S-MAIA instrument)

REP – To denote a repeated examination where original exam deemed to be unreliable by examiner.

Finally the folder must be converted to a single ZIP compressed archive.

h) Export folders must be named according to the labelling scheme in table 3 according to the data type contained within the folder. Refer to Section 8.1 of PM1 and the expressions for S-MAIA defined above.

Type of data	Labelling scheme
Certification data	MACUSTAR-Cert-StudyID-SiteID-Last name_first name-S_MAIA-xx
Study visits data	MACUSTAR-StudyID-SiteID-PatientID-Visit-S_MAIA
Resubmissions data	MACUSTAR-StudyID-SiteID-PatientID-Visit-S_MAIA-Resubmission

Table 3: Labelling scheme of Export Folders for submission to GRADE Reading Center.

i) Export files must be named according to the labelling scheme in table 4 according to the file type. Please note export files of resubmission data do not need special labelling.

j) Generate a single ZIP compressed archive by right clicking on the folder to be compressed. In the context menu select **Send To > Compressed (zipped) folder**. The ZIP-compressed folder will now be generated in the same directory as the source folder.

k) Results should be submitted to GRADE within 72 hours. Submit result to GRADE even if fixation losses are 30% or greater or if 95% BCEA exceeds 50deg^2.

File type	Labelling scheme for Mesopic(M) MAIA
PDF	MACUSTAR-StudyID-SiteID-PatientID-Visit-S_MAIA_M.pdf
Repeat exam PDF	MACUSTAR-StudyID-SiteID-PatientID-Visit- S_MAIA_M-REP.pdf
**File type**	**Labelling scheme for Scotopic(S) MAIA**
Red PDF	MACUSTAR-StudyID-SiteID-PatientID-Visit-S_MAIA_R.pdf
Repeat exam Red PDF	MACUSTAR-StudyID-SiteID-PatientID-Visit-S_MAIA_R-REP.pdf
**File type**	**Mesopic and Scotopic Raw Data**
TGZ file	MACUSTAR-StudyID-SiteID-PatientID-Visit-S_MAIA.tgz
**File type**	**Labelling scheme for Dark Adaptation Set Up Form**
DA set up form PDF	MACUSTAR-StudyID-SiteID-PatientID-Visit-DASF.pdf

Table 4: Labelling scheme of Export Files for submission to GRADE Reading Center.

4.3.10 Data backup

Each CS is responsible for a save data backup and storage. Length of storage should be in accordance to the study protocol, local rules and general licensing requirements.

To perform a backup, insert a USB or hard drive into any available USB port on the S-MAIA. Press the wheel symbol on the top right of the Start Up Screen to enter the settings page. Press 'Back up' to make a copy of all stored data on the S-MAIA. As the S-MAIA does not restrict access of any user to the full dataset, data can be deliberately or inadvertently deleted by any user.

4.3.11 End of test

a. Shut down the S-MAIA. Go to the start up screen and click on the power button in the top right corner of the screen.
b. An on screen message will appear when it is safe to shut down the S-MAIA. When safe to do so, turn off the power switch on the right side of the S-MAIA and replace the lens cap.
c. As the next MACUSTAR procedure requires the subject to be dark adapted, do not turn on the room lights.

4.4 Storage

Archive folders must have the study name, its content and the date of archiving explicitly stated. Study archive procedures will be provided by the Sponsor / designee at the end of the study.

5. LIST OF ATTACHMENTS

Attachment No.	Title
VF08-1	Microperimetry: Pearls and Pitfalls
VF08-2	Dark Adaptation Set Up Form
VF08-3	Application for Certification of Microperimetry and AdaptDx
VF08-5	Application for Certification of Photographers / Technicians for Microperimetry and AdaptDx
VF08-6	New Expert Exam 'MACUSTAR' grid placement in subjects with late AMD

EVICR.net EUROPEAN VISION CLINICAL RESEARCH	**Standard Operating Procedures**	macustar
SOP: VF09	**ADAPTDX ABSOLUTE THRESHOLD AND DARK ADAPTATION**	
Version: 3	Date: 2021-12-09	Replaces Version: 2
Related SOP(s): VF08 – S-MAIA Microperimetry and PM1 – Retinal Imaging – Standard Modalities		
Reasons for Revision	Added section under 4.2 Subject Information to stiplulate that any subject who has converted to Late AMD at a previous longitudinal study visit does not need to complete any AdaptDx procedures.	

Approval:	Name	Date	Signature
Author:	Alison Binns, CITY	2021-12-09	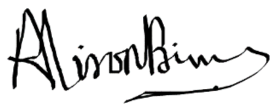
Reviewed:<br>(GCP compliance review)	Cecília Martinho, AIBILI	2021-12-09	
Authorised:	Frank Holz, UKB	2021-12-09	

Distribution

This SOP is part of the Manual of Study Procedures for the Clinical Study MACUSTAR and its distribution is performed by AIBILI in a controlled manner.

Table of Contents

1. purpose 41
2. policy/scope 41
3. STAFF TRAINING AND RESPONSIBILITIES 42
    3.1 Programming protocols on first use 42
    3.2 Training staff 43
4. PROCEDURE 45
    4.1 Equipment 45
        4.1.1 Equipment Maintenance 46
    4.2 Subject Information 46
    4.3 Technical Procedure 46

4.3.1 Considerations before starting tests 46
4.3.2 Setting up the AdaptDx 47
4.3.3 Preparing the subject 48
4.3.4 Subject information absolute threshold measurement 49
4.3.5 Description of procedure absolute threshold measurement 50
4.3.6 Reliability checks 51
4.3.7 Subject information rod intercept time measurement 52
4.3.8 Description of procedure rod intercept time measurement 53
4.3.9 Fixation 55
4.3.10 Reliability checks 55
4.3.11 End of the test 56
4.3.12 Labelling data and data exportation 56
4.3.13 Data backup 58
4.4 Storage 58
5. list of attachments 58

a) purpose

This document describes the procedures to perform absolute threshold and dark adaptation testing for the Clinical Study *"Development of novel clinical endpoints for interventional clinical trials with a regulatory and patient access intention in patients with intermediate age-related macular degeneration (AMD) – MACUSTAR"* (Protocol nº ECR-AMD-2017-13) to ensure that a uniform procedure is followed by all clinical sites (CS) participating in the study, in order to obtain comparable and reliable data, as according to International Conference on Harmonization Good Clinical Practice (ICH-GCP). This procedure will be performed on the study subjects according to the Clinical Study Protocol.

b) policy/scope

This standard operating procedure (SOP) will be used when performing dark adaptation examinations to ensure that all procedures are performed consistently. Adherence to this SOP will also ensure the proper treatment of participants, and that all data are available for future analysis.

This procedure describes the method for performing dark adaptation examinations on participants with age-related macular degeneration (AMD) and healthy controls for the

MACUSTAR cross-sectional and longitudinal studies. The AdaptDx is used to perform two separate but related procedures – a measurement of absolute threshold (the minimum amount of light detectable when fully dark adapted), and an assessment of the rate of rod dark adaptation (the 'rod intercept time'). The rod intercept time is the time taken for the eyes to reach a certain pre-defined threshold of log units attenuation after exposure to a bright flash of light.

Changes in the Clinical Study Protocol may lead to new versions of this SOP.

If the need for modification or withdrawal of the SOP or Attachment is identified, ensure that this information is reported in writing to the author of this SOP, Alison Binns (alison.binns.1@city.ac.uk) who will decide on the need of revision.

The Coordinating Centre, AIBILI, ensures the SOP is distributed to all that may be concerned.

SOPs must be written in English.

c) STAFF TRAINING AND RESPONSIBILITIES

d) Programming protocols on first use

Since the AdaptDx device does not have the MACUSTAR protocols pre-programmed, these must be programmed by the clinical site and saved before first use in the MACUSTAR Clinical Study. The following is the series of steps required to set up the Absolute threshold and Rod Intercept Time protocols for MACUSTAR Clinical Study on the AdaptDx system. **This will need to be carried out once only upon first using the device for the MACUSTAR Clinical Study.**

1) Switch on the device and wait several minutes to access the 'Home' Screen
2) From the home screen, tap on the "Settings" wheel in the top right-hand corner of the screen. This will bring up the "System Tools" menu.
3) On the left side of the screen will be a section "Database Management" and underneath this there will be a "Manage Protocol" option. Select this.
4) There will now be a list of all the test protocols saved in the AdaptDx database.
5) To add a new protocol, touch the "Add Protocol" button at the bottom of the screen.
6) On this screen, you will be able to enter a number of new protocol parameters:
    - Effective bleach
    - Starting stimulus
    - Stimulus duration
    - Stimulus location

    - Rest break
    - Maximum test duration
    - Terminate test at rod intercept? (YES/NO)
    - You can also change the protocol name at the top of the screen.

7) These parameters should be programmed according to the settings in Table 1. After entry of the desired parameters under the appropriate protocol name (as in Table 1) click the "Save and Done" button at the bottom of the screen to save and return to the «System Tools” screen.
8) Two new protocols are required, so the steps must be repeated for the second protocol in Table 1.
9) Once this is complete, please return to ‘manage protocol’ and select each of the two new protocols in turn so the parameters are shown on the screen, take a photograph of the screen, and e-mail to **alison.binns.1@city.ac.uk** and **h.dunbar@ucl.ac.uk** to confirm that everything is correct. This is important, as any error in programming the protocols will impact all data collected at the site.

Table 1: Outlining required settings for each new protocol. Note that the rod intercept time test should be terminated at the rod intercept, while the absolute threshold test should not.

	Absolute Threshold Test	Rod Intercept Test
Protocol Name	“Macustar Abs Thresh”	“Macustar Rod Int”
Effective bleach	20%	76%
Starting stimulus	3 log units	1 log units
Stimulus duration	200ms	200ms
Stimulus location	5 degrees inferiorly	12 degrees inferiorly
Rest break	15 seconds	15 seconds
Maximum test duration	4 mins	30 mins
Terminate test at rod intercept? (YES/NO)	NO	YES

e) Training staff

The Principal Investigator is responsible for ensuring that the appropriate personnel for performing AdaptDx Absolute threshold and Dark adaptation is identified and trained. All

technicians should read this procedure before starting the participation in the Study. Clinical Sites are recommended to have a minimum of 2 technicians in the investigational team for this procedure.

Technicians will be certified as follows:

11) Basic training in using the device will be provided by the manufacturer on installation.
12) Each technician will read this SOP and the AdaptDx pitfalls – att. VF09-1. Each technician will undertake the absolute threshold and rod intercept time tests on two subjects without retinal pathology (NOR1 and NOR2) and two subjects with AMD (AMD1 and AMD2). If absolute threshold data are collected which are considered to be unreliable by the technician, the test should be completed once straight after the first attempt (unless exposure to a bleaching light source has occurred, in which case a 45 minute period of dark adaptation is required prior to retesting). If rod intercept data are collected which are considered by the technician to be unreliable (based on criteria discussed in section 4.3.6 and 4.3.10), the technician should repeat the test once, allowing 30 mins of additional dark adaptation after the start of the previous test. Technicians should also complete one Dark Adaptation Set Up Form – att. VF08-2 for each subject tested. Each technician will also complete the AdaptDx Certification Examination via the electronic Certification Portal described below in 9).
13) Certification AdaptDx data should be exported and transferred to GRADE as described in section 4.3.12 via the GRADE Reading Centre Portal. Where a test was repeated, both the original and the repeat tests should be submitted.
14) Each technician will also complete the Application for Certification of Microperimetry and AdaptDx – att. VF08-3 for submission to GRADE. Forms can be filled out electronically or manually with black pen in capital letters to ensure legibility. General data related to study site and contact details of technicians are to be completed in Sections 1 and 2 respectively.
15) Tick Maculogix AdaptDx in Section 3 and record the serial number of the device and software version in use. Technicians must provide comments describing their experience of using the AdaptDx and any problems encountered. This report will state whether they consider the test results obtained from each subject to be of high quality and if not, what they consider to be the issues with the data. The report should also

outline each participant's refractive error and the refractive correction used for each participant.

16) The Application for Certification of Microperimetry and AdaptDx – att. VF08-3 and the Dark Adaptation Set Up Form – att. VF08-2 must be sent either by fax to +49 228 287 9014813 or scanned via email to macustar@grade-rc.de.

17) Prior to undertaking any certification procedures, technicians should complete the Application for Certification of Photographers / Technicians for Microperimetry and AdaptDx – VF08-5 recording the name and contact details for all technicians requesting to be certified. Tick boxes should be checked indicating the imaging modality(ies) each technician requests certification for. The Application for Certification of Photographers / Technicians for Microperimetry and Adapt Dx – VF08-5 must be sent either by fax to +49 228 287 9014813 or scanned via email to macustar@grade-rc.de.

18) On receipt of the Application for Certification of Photographers / Technicians for Microperimetry and AdaptDx – att. VF08-5, GRADE will issue a username and 2 passwords to each technician by email; one password for the Certification Portal and one password for the GRADE Reading Centre Portal. Please note, the two passwords will be different, but after initial sign in to each portal, technicians will be prompted to change their assigned password and may synchronise the 2 passwords if they wish.

19) Each technician will complete the AdaptDx Certification Examination via the Certification Portal (https://test.certification.macustar.eu). This examination must be passed with 100%.

20) Notice of certification will be provided by GRADE by email.

f) PROCEDURE

g) Equipment

This procedure requires the following equipment:

- AdaptDx adaptometer (Maculogix)
- Red plastic filter
- 1 watt, 660nm red LED diode torch (dim red torch)
- Hand held occluder

- Encrypted USB key
- Cotton pads
- Surgical tape
- Alcohol hand rub
- Alcohol wipes

h) Equipment Maintenance

The supplier recommendation for the equipment should be followed for maintenance. Only qualified and trained personnel should do service and repair.

i) Subject Information

All subjects, except those who have converted to Late AMD at a previous study visit should complete the Adapt Dx procedures.

Oral information regarding the examination that is being performed is given to the subject.

j) Technical Procedure

k) Considerations before starting tests

e) Subject identification

The MACUSTAR subject identification code should be inputted into all participant name fields. During the certification process, enter 'NOR1' and "NOR2" for the two people without retinal pathology datasets and "AMD1' or "AMD2" for the AMD datasets into all participant name fields during the certification process. Date of birth should be entered as 01-01-1950 for all subjects. Subjects' true birth dates should not be used.

f) Pupil dilatation

AdaptDx procedures will be carried out on a dilated pupil. Study eye will have been dilated prior to S-MAIA microperimetry (VF08) as follows:

1 drop of 1% tropicamide should be instilled into both eyes (in preparation for imaging both eyes after dark adapted testing). Subjects should be informed that pupil dilation will wear off in 4-6 hours and warned not to drive during this time.

Inform subjects to seek medical attention if they experience any discomfort during this time.

g) Dark Adaptation

The room in which the procedures take place should be completely dark. This includes blocking the output from any LEDs in the test room (e.g. from computers), unless the light output is dim and red. Light should be blocked from entering under closed doors, arrangements should be made to extinguish or block the output of safety lights. Only the dim red torch identified in section 4.1 should be used during the dark adapted procedures. The participant should have been dark adapted prior to beginning the scotopic microperimetry. They should be maintained in a dark adapted state throughout all AdaptDx procedures *i.e.* lights should remain off after scotopic microperimetry has been completed. A sign should be placed on the door informing personnel that dark adaptation is underway, to avoid it being inadvertently opened during dark adaptation. The door may be locked from the inside as further protection. If the participant is accidentally exposed to light then an additional period of 45 minutes dark adaptation must commence. Remind subjects they must not look at mobile phone, tablet or computer screens while dark adapted. Technicians must also not use mobile phone, tablet or computers while the subject is dark adapted.

h) Testing procedure

Only the study eye will be tested.

I) Setting up the AdaptDx

a) The room should remain completely dark throughout all set-up and testing to avoid light adapting the participant. The dim red torch may be used for moving around the room. Examiners must only use the red torch identified in section 4.1. The set-up of the device may take place before lights are extinguished at the start of microperimetry if preferred. Subjects and examiners must not use mobile phones, or other illuminated devices whilst the subject is dark adapted.

b) Remove the protective cover from the device. If not already in place, cover the touch screen of the AdaptDx with its red plastic filter. This should remain in place throughout dark adaptation and testing.

c) Switch on the AdaptDx and allow 5 minutes for computer to boot up and reach the home screen.

d) Select the participant from the list (search by MACUSTAR subject identification code in 'Search Name' field) or create a new patient and enter details (only anonymised MACUSTAR subject identification codes should be inputted into all ID and name boxes and date of birth should be given as 01-01-1950, avoiding giving any real dates of birth– see section 4.3.1(a)).

e) Press 'Test' button

f) Press down arrow next to 'protocol' box and select 'Macustar Abs Thresh'. The parameters of the 'Macustar Abs Threshold' protocol are described in Section 3.1 (Table 1).

g) Add an appropriate powered lens to the trial lens holder. This should be equal to the spherical equivalent of the subject's study eye refraction result measured during the same study visit, with an additional + 3.00 DS (to account for proximity of the target). Record the prescription used in the Dark Adaptation Set Up Form – att. VF08-2.

Example 1: a participant whose distance Rx was - 5.00 DS should be provided with a - 2.00 DS trial lens (-5.00 + 3.00 = -2.00DS).

Example 2: a participant whose distance Rx was + 2.00/- 0.50 x 70 should be provided with a + 4.75 DS trial lens (spherical equivalent distance
Rx = sphere + half cyl = + 1.75, + 3.00 = + 4.75 DS).

m) Preparing the subject

n) Wipe the chin rest, headrest, and response button with an alcohol pad. The subject will appreciate witnessing your attempts to provide them with a clean and sterile testing environment.

o) Seat the subject. Ensure that they are seated comfortably and adjust the table height to ensure that they can lean forward into the chin and headrest comfortably.

p) Remove any spectacles from the participant.

q) Tape a cotton wool pad to cover the non-study eye.

r) For absolute threshold measurement, we wish to prevent the participant from being exposed to the bleaching light, therefore hand the participant an occluder to hold over their test eye for the initial flash presentation.

s) Hand participant the response button.

t) Give the participant oral information.

n) Subject information absolute threshold measurement

a) After setting the participant up as described in Section 4.3.3, the participant should be advised of the first part of the test – absolute threshold measurement.

b) Oral information regarding the examination that is being to be performed is given to the subject as follows.

"In this next test I will be measuring your eye's sensitivity to dim lights by asking you to use a button to tell me when you can see a spot of light in the bowl in front of you.

With your chin on the chinrest and your forehead up against the bar, I would like you to look into the bowl where you will see a red spot of light. For the duration of this test I would like you to look directly at the red spot. Below the red spot, a larger circle of light will appear and disappear. The larger circle is blue-green in colour but may appear to be greyish when dim. Whenever you see this larger circle of light appear, please press the button in your hand. There may be long periods of time where you don't see this larger circle of light at all – don't worry, that is completely normal.

This first test will last for 4 minutes. Before the test starts, I will need you to shut your eye and place a cover over it. When I ask you to do so, please could you remove the cover, open your eye, and start looking at the red spot and pressing your response button whenever the blue-green larger circle of light appear. Do you understand?"

c) The participant should confirm they understand.

o) Description of procedure absolute threshold measurement

a) Select appropriate test eye on screen.

b) Ask the participant to sit forward and place their head on the chin rest (on the left side if testing the right eye and vice versa) and press their forehead against the forehead rest.

c) Adjust position of chinrest using button next to screen with illuminated red arrows to centre participant's pupil on cross hairs of eye tracker.

d) On screen, press on down arrow next to 'pupil size', and press 'automatic'. Ask participant to keep eyes wide open and press 'accept' if circle appears well matched to pupil outline. Use manual adjustment to modify pupil diameter if necessary.

e) Participant should shut their test eye and hold an occluder over it.

f) Instruct the participant:

"Now we will start the test. Please shut your eyes and keep the cover held over your left/right [*i.e.* study] eye"

THE COVERING OF THE STUDY EYE AT THIS STAGE IS IMPORTANT – IF THE PARTICIPANT'S EYE IS EXPOSED TO THE BLEACHING FLASH IT WILL NOT BE POSSIBLE TO MEASURE ABSOLUTE THRESHOLD WITHOUT A FURTHER PERIOD OF 45 MINUTES DARK ADAPTATION.

a) Press 'Start' on screen (a dim flash will occur immediately in the AdaptDx).

b) Immediately afterwards, say to participant:

"I would now like you to open your eyes, and to take the cover away from your right/left [i.e. study] eye. You should leave the eye patch on your other eye. Please now look at the red spot, and press your response button every time you see the large circle of light briefly appear beneath the central red light.

a) Threshold measurement will begin and will continue for 4 minutes.

b) The stimulus will get dimmer and dimmer when the participant reports seeing it using the button, and will then get brighter if they report not seeing it. You will see the presentations of the stimulus as a black spot on the graph on the screen. When a threshold has been set, a square will appear around the spot and the machine will beep indicating a 15 second rest period for the participant.

c) Instruct the participant:

"You now have a little break of about 10 seconds before the test begins again. Please keep your chin on the chinrest, but feel free to shut your eyes."

a) Watch the timer count down the rest period. When 3 seconds remain before the test resumes, there will be a second beep and the participant should be instructed to reopen their eyes, ready to continue the test.

b) During the test, you may need to periodically readjust the position of the chinrest to ensure that the pupil remains reasonably well centred on the cross-hairs in the video monitor.

c) Encourage the participant throughout that they are doing well, that it is completely normal not to see the spot for a while (if they haven't recently pressed the button), that they need to keep looking at the red spot etc.

d) When the test has completed, the machine will show the rod intercept time or, in the case of absolute threshold, 'rod intercept time not calculated'.

e) Allow the participant to sit back and relax after the test is completed, but keep room lights off.

f) If the participant does not meet the reliability indices (Section 4.3.6), or struggles to undertake the test and threshold results are erratic, the absolute threshold test can be repeated straight away without requiring a further period of adaptation. However, if the cover was not held in place when the bleaching flash was presented, or exposure to lights (other than threshold measuring stimuli) has occurred, a further 45-minute period of dark adaptation will be required before repeating the test.

g) Room lights should remain off at the end of the absolute threshold test.

p) Reliability checks

a. If, at the end of the test, the fixation errors exceed 30%, the test will need repeating. Save all results regardless of whether the reliability indices are met.

b. It is expected for <u>the absolute threshold test only</u> that rod intercept will not be calculated. For this test only a 'rod intercept time not calculated' error does not require repetition.

c. Threshold points should be fairly consistent across the 4 mins – for most patients this will be between around 3.0 and 4.5 log units. There may be some variability

in the first two minutes as the patient familiarises themselves with the test – this is acceptable.

d. For some subjects (especially younger controls and early AMD) it is possible that they will reach the lower ceiling of 5 log units – this does not indicate a need for repetition.

e. If the threshold points repeatedly hit the upper ceiling of around 0 - 1 log units, it suggests that the subject has a blind spot in the tested location, or that they do not understand the test. Unless the subject has late stage AMD, a repeat test is required following further instruction.

f. If the data are deemed unreliable, absolute threshold testing can be repeated immediately after the previous test (unless the eye has been exposed to light sources such as the bleaching flash of light – in which case an additional period of 45 minutes dark adaptation is required).

g. Additional guidance and troubleshooting tips can be found in the accompanying document 'How to perform a perfect AdaptDx' att. VF09 – 3.

h. In order to restart the test, go back to the home screen and follow steps in Section 4.3.5.

q) Subject information rod intercept time measurement

a) The participant should be given a 5-minute break after completing the absolute threshold test before starting the rod intercept time test – the room should remain in complete darkness for this period.

b) The participant set up is as for the absolute threshold test, except they must not hold the occluder over the test eye.

c) Ensure cotton wool eye patch is positioned over the non-study eye.

d) Hand participant the response button.

e) Ask the participant to sit forward and place their head on the chin rest (on the left hand side if testing the right eye and vice versa) and press their forehead firmly against the forehead rest.

f) Give participant oral instructions.

"We are going to do another test now which is very similar except that this time a bright flash of light will appear in front of you immediately before you start the test. This will be a bit like

the flash of a camera. This will dazzle you slightly and we will measure how long it takes for your eyes to readjust to the dark. Again, I would like you to look directly at the red spot for the duration of the test. When we start the test, a flash of light will be presented to your eye in the lower part of your vision – this will leave a bit of an after image. The blue-green spot of light will begin appearing and disappearing in the same location as the flash. This is a bit lower down than the spot appeared in the first test. Whenever you see the spot, you should press your button. The test will continue until your vision has fully recovered after being dazzled, up to a maximum of 30 minutes.

We will start with a practise test this time. Do you understand?"

g) The participant should confirm that they understand.

r) Description of procedure rod intercept time measurement

a) Select 'Macustar Rod Int' protocol on screen by touching the arrow next to the 'protocol' box. The parameters of the 'Macustar Rod Int' test are described in Section 3.1 (Table 1).

b) Select the appropriate test eye on the screen.

c) Ask the participant to sit forward and place their head on the chin rest (on the left hand side if testing the right eye and vice versa) and press their forehead against the forehead rest.

d) Adjust the position of chin rest by using the button next to screen with illuminated red arrows to centre participant's pupil on cross hairs of eye tracker.

e) On screen, press on down arrow next to 'pupil size', and press 'automatic'. Ask participant to keep eyes wide open and press 'accept' if circle appears well matched to pupil outline. Use manual adjustment to modify pupil diameter if necessary.

f) Instruct participant:

"This time we will have a practise test before we get started. Please look at the red spot all of the time. Have a few big blinks…. now open your eyes as wide as you can whilst looking at the red spot."

g) As soon as their eyes are wide open and correctly fixated, press the 'demo' button. At this point, a relatively dim flash of light will be presented to the eye, and then

the demo test begins. Remind the participant to press their button every time they see the blue-green spot appear in their lower visual field.

h) The stimulus will get dimmer and dimmer when the participant reports seeing it using the button, and will then get brighter if they report not seeing it. You will see the presentations of the stimulus as a black spot on the graph on the screen. When a threshold has been set, a square will appear around the spot and the machine will beep indicating a 15 second rest period for the participant.

i) Instruct the participant "You now have a little break of about 10 seconds before the test begins again. Please keep your chin on the chin rest, but feel free to shut your eyes."

j) Watch the timer count down the rest period. When 3 seconds remain before the test resumes, the device will beep again and the participant should be instructed to reopen their eyes, ready for the test to resume.

k) Continue with the demo until you are confident that the participant knows what to do, and until they have had at least two threshold points set. Select 'Quit'.

l) Ask the participant:

"Are you happy to undertake the test now, or do you have any questions?"

Once any queries have been resolved, say that you will begin the main test.

m) Instruct the participant:

"Now we will start the proper test. This will be just like the practise run, but will go on a bit longer and the flash of light at the beginning will be brighter than when we practised. It's really important that you look at the red spot all of the time. Have a few big blinks…. now open your eyes as wide as you can whilst looking at the red spot."

n) ACCURATE FIXATION AND A FULLY OPEN EYE DURING THE BLEACHING FLASH IS VITAL – if the participant is not correctly fixating at this point, the test will not work and the participant must be dark adapted for a further 30 minutes before re-attempting the test.

o) As soon as their eyes are wide open and correctly fixated, press the start button. At this point, a bright flash of light will be presented to the eye, and then the main test begins. Remind the participant to press their button every time they see the blue-green circle appear in their lower visual field.

p) Threshold measurement will begin as in the 'Demo test'.

q) During the test, you may need to periodically readjust the position of the chinrest to ensure that the pupil remains reasonably well centred on the cross-hairs in the video monitor.

r) This process will continue for up to 30 minutes (the test will automatically stop when the rod intercept has been reached or after 30 minutes).

s) Encourage the participant throughout that they are doing well, that it is completely normal not to see the spot for a while (if they haven't recently pressed the button), that they need to keep looking at the red spot etc.

t) When the test has completed, the machine will show the rod intercept time or 'rod intercept time not calculated' (see Section 4.3.10).

u) Allow the participant to sit back and relax after the exam.

v) If the result is acceptable, the lights may be switched on (gradually, to avoid dazzling the dark adapted participant).

w) If reliability indices are not met (Section 4.3.10) then the participant should be kept in the dark at this stage.

s) Fixation

a) You will see the participant's eye on the screen before you throughout the test. If you notice any deviations in gaze, remind the participant to keep focussing on the red spot.

b) The AdaptDx will report if it suspects a fixation error (due to an unexpected change in threshold). A fixation error is indicated when a measured threshold point is shown as an unfilled instead of a filled circle, and the device begins measuring the next threshold point without giving the participant a rest break. In this case, remind the participant to keep focussing on the red spot.

t) Reliability checks

a) If, at the end of the test, the fixation errors exceed 30%, or there is an error message saying 'rod intercept time not calculated', the test will need repeating. Save all results regardless of whether the reliability indices are met.

b) Threshold points will usually begin at between 1-2 log units, and decrease systematically to below 3 log units. If they begin below 3 log units or drop there

immediately, this indicates that the bleach was not effectively delivered and the test should be repeated.

c) Rod intercept time testing can be repeated 30 minutes after the start of the previous test if data are deemed unreliable. For this washout period, the participant should be left to continue dark adapting.

d) Additional guidance and troubleshooting tips can be found in the accompanying document ‘How to perform a perfect AdaptDx’ att. VF09 – 3.

e) In order to restart the test, go back to the home screen and follow steps in Section 4.3.8.

u) End of the test

a) Return to home screen. Test results will save automatically on hard drive.

b) Turn off computer.

c) Instil an additional drop of 1% tropicamide to each eye at the end of the procedure in preparation for imaging tests.

v) Labelling data and data exportation

a. Results should be backed up to the encrypted USB key (PDF and XML file) and submitted to GRADE within 72 hours. Submit result to GRADE even if fixation errors exceed 30% or ‘rod intercept time not calculated’ error is obtained. If test is repeated, submit both with comments on reason for repetition.

b. If test is repeated, record most reliable result on the eCRF.

c. In order to export data, ensure encrypted USB key is in USB port.

d. Select subject on home screen and press ‘show prior tests’.

e. Select relevant (up to 2 at one time) tests.

f. Press ‘print to PDF’

g. Repeat process for remaining tests if necessary.

h. Return to home screen and go to settings wheel in top R corner.

i. Under ‘System settings’ press ‘set print destination’.

j. Set to ‘Print to XML’ (you can also use «set print destination» to switch back to PDF printing from XML printing).

k. Return to home screen, select participant again; select relevant (up to 2) tests again.

l. Touch ‘Print to XML’.

m. Repeat process for remaining tests if necessary.

n. Prior to submission to GRADE, all AdaptDx files (PDF and XML) must be renamed and saved in appropriately named folders according to Section 8 of the Procedure Manual 1 (PM1) – Retinal Imaging – Standard Modalities. GRADE may reject submissions with incorrectly labelled files or folders. Rejected data can be resubmitted after correction. File labelling expressions are described in Section 8.1 of the PM1 where the following expression should be used for AdaptDx:

   Modality – Use the following abbreviations for indicating modalities:

   ADX (= Maculogix Adapt Dx)

   ABSTHRESH-ADX =(Adapt Dx Absolute Threshold)

   RIT-ADX (= Adapt Dx Rod Intercept Time)

   DASF (=Dark Adapted Set Up form)

   REP – To denote a repeated examination where original exam deemed to be unreliable by examiner.

Finally, the folder must be converted to a single ZIP compressed archive.

o. Export folders must be named according to the labelling scheme in Table 2 according to the data type contained within the folder.

Table 2: Labelling scheme of Export Folders for submission to GRADE Reading Center.

Type of data	Labelling scheme
Certification data	MACUSTAR-Cert-StudyID-SiteID-Last name_first name-ADX-xx
Study visits data	MACUSTAR-StudyID-SiteID-PatientID-Visit-ADX
Resubmissions data	MACUSTAR-StudyID-SiteID-PatientID-Visit-ADX-Resubmission

p. Export files must be named according to the labelling scheme in Table 3 according to the file type. Please note export files of resubmission data do not need special labelling.

Table 3: Labelling scheme of Export Files for submission to GRADE Reading Center.

File type	Labelling scheme for Absolute Threshold Exam
PDF	MACUSTAR-StudyID-SiteID-PatientID-Visit-ABSTHRESH-ADX.pdf
Repeat exam PDF	MACUSTAR-StudyID-SiteID-PatientID-Visit- ABSTHRESH -ADX-REP.pdf

XML	MACUSTAR-StudyID-SiteID-PatientID-Visit-ABSTHRESH- ADX.xml
Repeat exam XML	MACUSTAR-StudyID-SiteID-PatientID-Visit-ABSTHRESH-ADX-REP.xml
**File type**	**Labelling scheme for Rod Intercept Time Exam**
PDF	MACUSTAR-StudyID-SiteID-PatientID-Visit- RIT-ADX.pdf
Repeat exam PDF	MACUSTAR-StudyID-SiteID-PatientID-Visit-RIT-ADX-REP.pdf
XML	MACUSTAR-StudyID-SiteID-PatientID-Visit-RIT-ADX.xml
Repeat exam XML	MACUSTAR-StudyID-SiteID-PatientID-Visit-RIT-ADX-REP.xml

q. Generate a single ZIP compressed archive by right clicking on the fold to be compressed. In the context menu select **Send To > Compressed (zipped) folder**. The ZIP-compressed folder will now be generated in the same directory as the source folder.

w) Data backup

Each CS is responsible for a safe data backup and storage. Length of storage should be in accordance with the study protocol, local rules and general licensing requirements. Data should be backed up in PDF and XML format onto the memory stick in the back of the AdaptDx device (see Section 4.3.12 for details on how to export and save data).

x) Storage

Archive folders must have the study name, its content and the date of archiving explicitly stated. Study archive procedures will be provided by the Sponsor / designee at the end of the study.

y) list of attachments

Attachment No.	Title
VF09-1	AdaptDx pitfalls
VF08-2	Dark Adaptation Set Up Form
VF08-3	Application for Certification of Microperimetry and AdaptDx
VF08-5	Application for Certification of Photographers / Technicians for Microperimetry and AdaptDx
VF09-3	How to perform a perfect AdaptDx

References

1. Steinberg JS, Saßmannshausen M, Pfau M, et al. Evaluation of two systems for fundus-controlled scotopic and mesopic perimetry in eye with age-related macular degeneration. *Translational Vision Science and Technology*. 2017;6(4):7. doi:10.1167/tvst.6.4.7

2. Curcio CA, Medeiros NE, Millican CL. Photoreceptor loss in age-related macular degeneration. *Investigative Ophthalmology and Visual Science*. 1996;37(7):1236-1249. http://www.ncbi.nlm.nih.gov/pubmed/8641827

3. Nebbioso M, Barbato A, Pescosolido N. Scotopic microperimetry in the early diagnosis of age-related macular degeneration: Preliminary study. Mizota A, ed. *BioMed Research International*. 2014;2014:671529. doi:10.1155/2014/671529

4. Welker SG, Pfau M, Heinemann M, Schmitz-Valckenberg S, Holz FG, Finger RP. Retest Reliability of Mesopic and Dark-Adapted Microperimetry in Patients With Intermediate Age-Related Macular Degeneration and Age-Matched Controls. *Investigative Ophthalmology & Visual Science*. 2018;59(4):AMD152-AMD159. doi:10.1167/iovs.18-23878

5. Jackson GR, Scott IU, Kim IK, Quillen DA, Iannaccone A, Edwards JG. Diagnostic Sensitivity and Specificity of Dark Adaptometry for Detection of Age-Related Macular Degeneration. *Investigative Opthalmology & Visual Science*. 2014;55(3):1427. doi:10.1167/iovs.13-13745
